# Electrically Stimulated Tunable Drug Delivery From Polypyrrole-Coated Polyvinylidene Fluoride

**DOI:** 10.3389/fchem.2021.599631

**Published:** 2021-02-05

**Authors:** Solaleh Miar, Joo L. Ong, Rena Bizios, Teja Guda

**Affiliations:** Department of Biomedical Engineering and Chemical Engineering, University of Texas at San Antonio, San Antonio, TX, United States

**Keywords:** electrosensitive, drug delivery, polypyrrole (PPy), polyvinylidene fluoride (PVDF), nerve growth factor, basic fibroblast growth factor (bFGF)

## Abstract

Electrical stimulus-responsive drug delivery from conducting polymers such as polypyrrole (PPy) has been limited by lack of versatile polymerization techniques and limitations in drug-loading strategies. In the present study, we report an *in-situ* chemical polymerization technique for incorporation of biotin, as the doping agent, to establish electrosensitive drug release from PPy-coated substrates. Aligned electrospun polyvinylidene fluoride (PVDF) fibers were used as a substrate for the PPy-coating and basic fibroblast growth factor and nerve growth factor were the model growth factors demonstrated for potential applications in musculoskeletal tissue regeneration. It was observed that 18-h of continuous polymerization produced an optimal coating of PPy on the surface of the PVDF electrospun fibers with significantly increased hydrophilicity and no substantial changes observed in fiber orientation or individual fiber thickness. This PPy-PVDF system was used as the platform for loading the aforementioned growth factors, using streptavidin as the drug-complex carrier. The release profile of incorporated biotinylated growth factors exhibited electrosensitive release behavior while the PPy-PVDF complex proved stable for a period of 14 days and suitable as a stimulus responsive drug delivery depot. Critically, the growth factors retained bioactivity after release. In conclusion, the present study established a systematic methodology to prepare PPy coated systems with electrosensitive drug release capabilities which can potentially be used to encourage targeted tissue regeneration and other biomedical applications.

## Introduction

Bioelectrical stimuli have been extensively harnessed to stimulate cellular activity and promote tissue regenerative response within several tissue types including bone (Porter et al., 2009), nerve (Ghasemi-Mobarakeh et al., 2011), skeletal muscle (Donnelly et al., 2010) and cardiac muscle (Radisic et al., 2004). Poly-pyrrole (PPy), a bio-compatible polymer is an ideal candidate to elicit such responses because of its inherent electrical conductivity and ability to be used as a biomaterial scaffold substrate. These attributes of PPy have been widely demonstrated to stimulate regeneration of multiple tissues including bone (Sajesh et al., 2013; Song et al., 2016), nerve (Stewart et al., 2015; Thunberg et al., 2015), skeletal muscle (Gilmore et al., 2009; Browe and Freeman, 2019), and cardiac muscle (Kai et al., 2011; Spearman et al., 2015). While the electro-activity of PPy has been the primary feature leveraged for stimulating cell and tissue function, an ancillary advantage that PPy offers is the potential for simultaneous drug/growth factor delivery induced by electrical stimulation. Electrical conductivity is introduced within PPy materials by including negatively charged dopants, which enable the conduction of charge along the polymer backbone under stimulation. Dopants thus offer a most suitable site for tethering drugs intended for delivery under electrical stimulation. Stimulus-responsive drug delivery from PPy; however, has been limited (Geetha et al., 2006) by the availability of suitable dopants (Green et al., 2008) within the system, as well as by drug molecular weight and charge (Miller, 1988). In order to circumvent these limitations, biotin has been previously investigated as a dopant to be conjugated to form a drug complex for further drug release in electro-deposited PPy (George et al., 2006).

Electrodeposition is an efficient method to synthesize PPy (Asavapiriyanont et al., 1984). However, the product of electrochemical polymerization synthesized on the working electrode is a thin film with poor mechanical properties and handleability (Diaz and Hall, 1983; Guimard et al., 2007). These limitations constrain the use of electrochemical polymerization in the preparation of tissue engineering scaffolds and hamper further translational application due to the difficulty in removal of coatings from the electrodes. Chemical oxidation, another widely used technique enables the production of PPy in the form of thin polymeric films (Lu et al., 1998), incorporation in composite scaffolds (Lee et al., 2009; Pelto et al., 2013) and as microparticles (Ramanaviciene et al., 2007). Despite the potential for drug delivery via the incorporation of growth factors or other drugs as doping agents during synthesis of PPy by chemical oxidation, applications in controlled drug delivery remain relatively unexplored. Past efforts associated with chemical oxidation synthesis of PPy have established incorporation but not controlled release of heparin (Meng et al., 2008) or the uncontrolled release of incorporated drugs by diffusive passive leakage (Bay et al., 2002; Fonner et al., 2008; Balint et al., 2014). PPy coatings deposited on polymeric substrates by chemical oxidation methods have been previously investigated for neural tissue engineering, but growth factors have yet to be incorporated (Jin et al., 2012; Shrestha et al., 2018) and parameters for successful electrical-stimulation-based delivery have yet to be identified (Cho and Borgens, 2011).

In the present study, we hypothesize that a low temperature *in situ* polymerization method (Merlini et al., 2014b), which involves sodium *p*-toluenesulfonate (SPTS) as a permanent doping agent along with biotin as a co-dopant (George et al., 2006), will allow for *in situ* polymerization of PPy coatings capable of growth factor incorporation for stimulus responsive delivery on a variety of substrates. This *in situ* polymerization method is developed to demonstrate the capability to coat Polyvinylidene fluoride (PVDF) microfibers with growth factor conjugated PPy and further, to generate a stimulus-responsive, growth factor release capable, electroactive substrate for tissue engineering applications.

PVDF is an electroactive polymer with excellent piezoelectric properties compared to other biocompatible polymers (Ribeiro et al., 2015); however, limitations with regards to both drug incorporation as well as diffusion-based or stimulus-responsive release of drugs, restrict its applications for direct use as an substrate for electrically stimulated tissue engineering applications (Ribeiro et al., 2015). Chemical oxidation using FeCl_3_ as the initiator has been used previously to coat randomly oriented PVDF fibers with PPy (Rao et al., 2013); and the composite fibers have been evaluated for applications in energy harvesting (Merlini et al., 2014b). The conductivity of PPy-coated PVDF fibers synthesized through *in situ* polymerization has also been previously investigated (Merlini et al., 2014a). However, no studies to date have assessed piezoelectric PVDF fibers, coated *in situ* with PPy to develop composite materials for stimulus responsive drug release for biomedical applications. The development of such an approach facilitates the enhancement of the surface properties of PVDF and potentially improving bioactivity; while the piezoelectric (PVDF) – electroconductive (PPy) material combination allows for drug delivery that is exquisitely sensitive to applied electrical stimuli.

In the present study, we use an electric field as the stimulus to investigate the sensitivity of controlled, conjugated growth-factor release from the PPy coating (using *in situ* polymerization) on PVDF fibers. Basic Fibroblast Growth Factor (bFGF) and Nerve Growth Factor (NGF) are used as model drugs to illustrate electrical stimulation responsive controlled growth factor release, and post-release bioactivity. bFGF influences satellite cells proliferation and differentiation into mature myotubes (Guthridge et al., 1992) while NGF improves innervation in skeletal muscle (Slack et al., 1983) in addition to promoting the survival and differentiation of neurons (Kurata et al., 2013). Both growth factors can also be biotinylated without significantly affecting its bioactivity (Rosenberg et al., 1986; Pieri and Barritault, 1991), which is essential for the proposed modality of controlled drug delivery.

## Material and Methods

### Synthesis of PPy-Coated PVDF-Electrospun Fibers

Polyvinylidene fluoride (PVDF) electrospun fibers were used as a model substrate for deposition of polypyrrole (PPy) coating in which biotin was incorporated via chemical polymerization. Electrospun PVDF fibers were collected on a rotating drum following a technique adapted from the literature (Yee et al., 2007; Cozza et al., 2013). Briefly, a 20% PVDF solution was prepared by dissolving PVDF pellets (molecular weight: 275,000) in dimethylformamide (DMF) and acetone (5:4 v/v ratio). The polymer-containing solution in a syringe (needle gauge: 18) was placed in the syringe pump (Pump 11 Elite, Harvard Apparatus, Holliston, MA) of the electrospinning set-up. In order to obtain aligned fibers, the rotating drum of the electrospinning set-up was placed 30 cm away from the polymer-containing syringe; and the liquid was delivered at the flow rate 0.6 ml/h. Electrospinning was accomplished at an applied voltage of 20 kV and a rotating drum speed of 400 rpm. The PVDF fibers thus obtained were then maintained under vacuum for 24 h (to accelerate solvent evaporation).

Distilled pyrrole (15 mM) was mixed with an aqueous solution (1 mM) of sodium *p*-toluenesulfate (SPTS; 95%) and biotin (50 mM) in pure ethanol under nitrogen. In order to optimize the coating process, the PVDF fibers were soaked in this solution for 30 min at room temperature followed by ultrasonication for 5 min (to remove trapped air bubbles), prior to addition of ferric chloride solution (4 mM), and continuous shaking at 4 °C for preselected time intervals of 1, 6, 12, 18 and 24 h to produce various PPy coatings. The polymerization process was terminated at the prescribed time points by rinsing the coated PVDF fibers with copious amount of a water: ethanol (30:70 v/v) solution to remove unreacted chemicals and PPy debris. All chemicals and reagents used in the aforementioned processes were purchased from Sigma- Aldrich (MO, United States).

### Morphology of PPy-Coated PVDF-Electrospun Fibers

Scanning Tunneling Electron Microscopy (STEM) model S5500 (Hitachi High-Tech, Schaumburg, IL) was used to examine the morphology of the pure PVDF electrospun fibers and the five groups of PPy-coated PVDF fibers after polymerization for the various preselected time intervals. All specimens were coated with silver-palladium and imaged under 30 kV applied voltage at 3,000x and 15,000x magnifications. The collected micrographs were then analyzed to quantify orientation distribution of electrospun PVDF fibers, before and after PPy coating, using ImageJ (v1.8.0, NIH Image, Bethesda, MD) to evaluate the impact of PPy polymerization on the alignment of the PVDF fibers.

### Wettability of PPy-Coated PVDF-Electrospun Fibers

The hydrophilicity of electrospun fiber mats (both PVDF and the five groups of PPy coated PVDF fibers) was determined using the contact angle sessile drop method at room temperature. A water droplet (2 µL in volume) was introduced on the flat surface of mats and the contact angle was recorded using the VCA Optima System (AST Products Inc., Billerica, MA). In addition, the impact of PPy debris, produced during the polymerization, on wettability of the PPy coated PVDF fibers was studied by sonication (15 min at room temperature) of the samples to remove the debris, followed by comparison of the contact angle between the pre-sonication and post-sonication PVDF and PPy coated PVDF fiber samples. This analysis was conducted for four samples per group to quantify the wettability properties of the samples.

### Composition Characterization of PPy-Coated, PVDF Electrospun Fibers

Uncoated PVDF fibers, PPy-coated PVDF fibers doped with biotin, and PPy-coated PVDF fibers without biotin were examined using Attenuated Total Reflection Fourier Transform IR (ATR-FTIR; Tensor 27; Bruker Optics, Billerica, MA) in order to evaluate the relative fraction of the polar *β* phase (β ratio) of the PVDF and to confirm incorporation of biotin, a co-doping agent, in the polymerized PPy. The *β* ratio of PVDF electrospun fibers was calculated using the equation: $f\left(\beta \right)=\frac{A_{\beta }}{1.26\;A_{\alpha }+A_{\beta }}$, where A_α_ is the absorbance for the infrared absorption band at 530 cm^−1^ (representing CF_2_ bending of the α-phase) and A_β_ is the absorbance for the infrared absorption band at 840 cm^−1^ (representing CH_2_ bending of the β -phase) (Yee et al., 2007). The absorbance values A_α_ and A_β_ were calculated from the FTIR spectra (acquired at a resolution of 4 cm^−1^ and a scan number of 16) for the respective samples.

### Tensile Elastic Properties

Samples were dried in a lyophilizer overnight and cut into 10 mm × 5 mm sizes (n = 6 samples per group). Uniaxial elastic modulus was measured in the direction aligned with fiber orientation using a UStretch testing system (CellScale Biomaterials Testing, Ontario, Canada) at room temperature. The length between the clamps was set to 5 mm, and the thickness of each sample was measured using a micrometer prior to testing. Samples were stretched uniaxially to failure and the slope of the load-displacement graph was used to calculate the elastic modulus.

### Microindentation

The local elastic modulus of the fibers was measured with a microindenter (Piuma Chiaro, Optics11, Amsterdam, The Netherlands). A probe with a tip radius of 21 µm and cantilever spring constant of 3.88 N/m was used to measure the elastic modulus of the electrospun fibers. Dry samples (n = 6 specimen per group) were indented using mapping mode at room temperature; the scan size was 10 spots spaced 25 μm apart along a straight line per sample. The loading and unloading force-displacement curves were acquired during each indentation process and the Oliver-Pharr (Oliver and Pharr, 1992) model was used to calculate tip-substratum area of contact and determine the elastic modulus from the initial linear region of the unloading curve by then assuming Hertzian contact between the indenter and substrate (Casey et al., 2017).

### Characterization of Electrical Conductivity

Dry samples (n = 3 per group) for each duration of PPy coating were compressed into films to remove the air between the fibers. The voltage and current of the films were then measured three times using a four probe machine (Silicon Valley Science Labs, Oak Ridge, TN) at room temperature. Conductivity (σ measured in Ω^−1^. cm^−1^) was calculated using the equation $\sigma =\frac{1}{\left(\text{R}\times \text{T}\right)}$ where R is the surface resistance (Ω/sq) and T is the thickness (cm) as previously reported (Harjo et al., 2020).

### Biotin Incorporation and Stability in the PPy Coating on PVDF Fibers

In order to determine incorporation biotin into, and subsequent stability of biotin within the PPy coating; the PPy-coated PVDF fibers were maintained in phosphate-buffered saline (PBS) at 37 °C for two weeks. Samples were extracted from PBS after 0, 7 and 14 days of incubation and treated with Alexa Fluor™ 350 conjugated streptavidin (Thermofisher, Waltham, MA,USA) for 15 min, rinsed three times with PBS, and imaged using a Lionheart FX Automated microscope (BioTek Instruments Inc., Winooski, VT). Biotin incorporated in the PPy coating binds covalently to Streptavidin and the fluorescent intensity of the conjugated Alexa Fluor™ 350 in PPy coating was then used as an indicator of residual biotin in the PPy coating. The fluorescent intensity from the microscopy images was quantified using CTAn software (v1.17, Bruker BioSpin, Billerica, MA) to analyze biotin stability in the PPy coating over 14 days. This analysis was performed for six samples per group (time point).

### Voltage-Sensitive Release of Drug Carrier From PPy-Coated PVDF-Electrospun Fibers

The electrosensitivity of drug delivery from the PPy-coated PVDF fibers, was determined at various voltage regimes (120, 200, 280 mV/mm, applied at 1 Hz). For this purpose, C-Dish™ (IonOptix, Amsterdam, The Netherlands) carbon electrode-equipped culture plates were used to apply electrical stimulation as programmed by C-Pace EM (IonOptix, Amsterdam, The Netherlands). The PPy coated PVDF fibers were first maintained in streptavidin horseradish peroxide (HRP) (0.2 mg/ml; Thermofisher, MA, United States) solution at room temperature for 15 min and rinsed three times with PBS before being placed in the C-Dish™ plates containing fresh PBS (1 ml) and then exposed to electric pulses of 120, 200, 280 mV/mm (at 1 Hz frequency) applied to each sample for 30 min. The supernatant from these samples was collected every 10 min; and replaced with fresh PBS. The absorbance of the released streptavidin HRP in the aliquot was later measured using a Multi-Mode plate reader (Synergy 2, Biomek Instruments Inc., Winooski, VT). This analysis was performed for six samples per electrical stimulation level and time point.

The sustainability of the PPy-coated PVDF fibers as a drug delivery platform over time was determined by maintaining the PPy-coated PVDF fibers in streptavidin Alexa Fluor™ 350 conjugate for 15 min and then rinsing with PBS three times. The intensity of fluorescence of the residual streptavidin Alexa Fluor™ 350 conjugate in the PPy-coating was examined before and after exposure to electrical stimulation (200mV/mm; 1 Hz) using the C-Dish™ electrodes. The PPy-coated PVDF fibers were imaged using the Lionheart FX Automated microscope and the fluorescent intensity of the collected images were quantified using CTan software as detailed in *Tensile Elastic Properties*.

### Voltage-Induced Growth Factor Release From PPy-Coated PVDF-Electrospun Fibers

**FIGURE 1:**
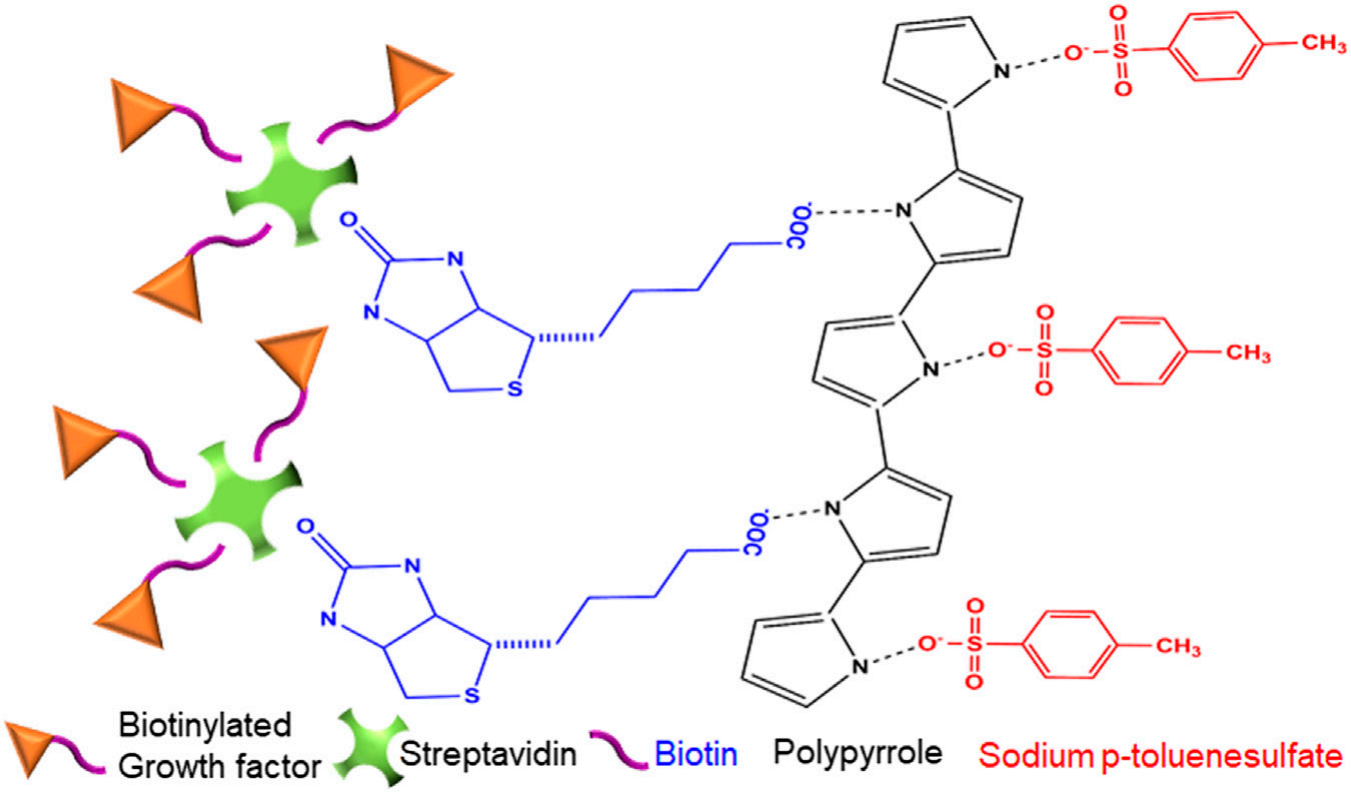
Schematic representation of electrosensitive drug delivery coating. Illustration of the chemical structure of the PPy coating containing sodium *p*-toluenesulfate (SPTS) and biotin as the primary and secondary doping agents. Streptavidin, with four potential binding sites, covalently binds to the biotin incorporated in the PPy-coating at one site and up to three biotinylated growth factors at the other sites, to function as the drug-complex carrier.

PPy-coated PVDF fibers were immersed in Streptavidin HRP solution (0.2 mg/ml) at room temperature for 15 min, then rinsed three times with PBS (to remove unconjugated streptavidin), and successively treated with biotinylated basic Fibroblast Growth Factor (bFGF; ACROBiosystems Inc., Newark, DE, USA) solution at 40 ng/ml in deionized water for 30 min, and again rinsed three times with PBS. A similar procedure was used for loading a solution of biotinylated Nerve Growth Factor (NGF, Alomone Labs, Jerusalem, Israel) at a concentration of 120 ng/ml in deionized water into a different group of PPy-coated PVDF fibers. Figure 1 illustrates the final schematic of the chemical structure of drug carrier platform containing streptavidin conjugated to biotinylated growth factor and anchored to the biotin incorporated within the PPy coating on the PVDF fibers.

The release profiles of 1) bFGF and 2) NGF were investigated under the applied electrical stimulation (200 mV/mm; 1 Hz) in the C-Dish™ plates in 1 ml of PBS. The PBS containing the released growth factors (either bFGF or NGF) was collected after 5 min of exposure to electrical stimulation and samples were equilibrated in fresh PBS for 5 min prior to the next electrical stimulation at the same settings. This sequence was repeated three times to estimate reproducibility and efficacy of the drug delivery platform, this was performed for four technical replicates per growth factor. The released growth factors were quantified using Enzyme Linked Immunosorbent Assays (ELISAs). Since the drug complex contains streptavidin, which can interfere with conjugation of the biotinylated antibodies to the growth factors, a modified ELISA technique was used to determine the released growth factors (Lakshmipriya et al., 2016). The reagents used for conducting the ELISAs in the present study were purchased from Thermofisher (Waltham, MA, United States).

### Bioactivity of Released Growth Factors From PPy-Coated PVDF-Electrospun Fibers

The biological activity of the released bFGF was determined using *in vitro* cell viability and proliferation assays. For this purpose, BALB/3T3 cells (ATCC #CCL163, Manassas, VA, USA), were cultured in Dulbecco's Modified Eagle's Medium, containing 10% of Fetal Bovine Serum (FBS), and 1% antibiotics (Ryu et al., 2007). Cells were initially cultured in tissue culture flasks, and were passaged using trypsin once they reached 80% confluence (Ryu et al., 2007). For the viability and proliferation assay, 5,000 cells were seeded in individual wells of a 96-well tissue culture treated wellplate. The proliferation of the BALB/3T3 cells was determined after exposure to recombinant human bFGF (positive control), biotinylated bFGF, and the bFGF released via electrical stimulation from the PPy-coated PVDF fibers using the CellTiter 96® AQueous Non-Radioactive Cell Proliferation Assay, for six biological replicates per group. The recombinant human bFGF and biotinylated bFGF were matched in concentration to the measured level of bFGF released by electrical stimulation.

The bioactivity of the released NGF was determined via an *in vitro* assay using PC12 (ATCC #CRL-1721, Manassas, VA, United States) cells. These cells were cultured using established protocols (Robinson and Stammers, 1994) in RPMI-1640 Medium, containing 10% heat-inactivated horse serum, 5% of FBS, and 1% antibiotics were cultured in tissue-culture flasks coated with Poly-l-lysine solution (0.1% (w/v)). Cells were initially cultured in tissue culture flasks, and were passaged using trypsin once they reached 80% confluence (Robinson and Stammers, 1994). 3.6 × 10^4^ PC12 cells were seeded onto individual wells of a poly-l-lysine-coated 96-well plate. The cells were then exposed to NGF (positive control), biotinylated NGF, and NGF released from PPy coated PVDF fibers by electrical stimulation for 20 h before their proliferation was determined using the CellTiter 96® Aqueous Non-Radioactive Cell Proliferation Assay (Zhu et al., 2015), for six biological replicates per group. The NGF and biotinylated NGF were matched in concentration to the measured level of bFGF released by electrical stimulation.

The proliferation behavior of the BALB/3T3 exposed to the bFGF and PC12 cells exposed to the NGF were reported as a measure of the bioactivity of released growth factors. Cell culture related supplies were purchased from Thermofisher (MA, USA) otherwise mentioned.

### Statistical Analyses

All numerical data are reported as the average ± the standard error of the mean. Material characterization data was acquired for four technical replicate samples per group (PVDF and the five durations of PPy polymerization); for six technical replicates per voltage in terms of drug complex release and biotin stability; for four technical replicate samples per growth factor (bFGF and NGF) in terms of voltage stimulated release and for six biological replicates per cell type for the bioactivity assays. The orientation of the fibers was determined using z-scores as the statistical metric in MedCalc (v19.4.1, MedCalc Software Limited, Oostende, Belgium). Significant differences in the numerical data for the material characterization analyses as well as for the drug release and bioactivity assays were identified using one-way Analysis of Variance (ANOVA), followed by Tukey’s test for *post-hoc* determination. Statistical analysis was conducted using SigmaPlot (v13, Systat Software Inc., San Jose, CA) and Prism (v9, GraphPad Software, San Diego, CA). A value of *p* < 0.05 was considered statistically significant.

## Results

### Morphology of PPy-Coated PVDF-Electrospun Fibers

**FIGURE 2:**
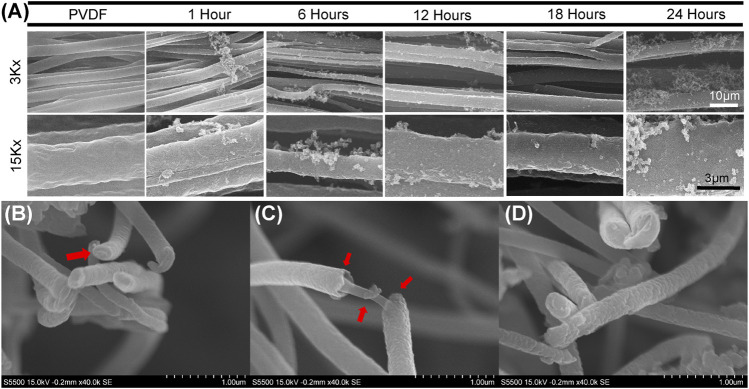
Scanning electron micrography of PPy coating morphology on aligned PVDF fibers **(A)** Morphology of uncoated, aligned PVDF fibers and PPy-coated PVDF fibers by PPy polymerization for 1, 6, 12, 18, 24 h. Scanning electron micrographs at two different magnifications (3,000x and 15,000x) show coating stabilization and some PPy debris accumulation around PVDF fibers over time **(B,C and D)** Micrographs of PVDF electrospun fibers after PPy coating for 18 h **(B and D)** Cross sections of fibers with PVDF fibers not distinguishable from the PPy coating, while **(C)** imaging along the length of fibers shows formation of the core-shell structure after coating.

Scanning Tunneling Electron Microscopy (STEM) demonstrated that PPy polymerization resulted in PPy fibers and debris deposited haphazardly around the PVDF fibers in the initial hours (after 1, 6, and 12 h in Figure 2) of polymerization until it formed a uniform coating around the PVDF fibers with minimal randomly oriented debris after 18 h of polymerization (Figure 2). However, random clusters and debris were again observed after 24 h of polymerization. While some PPy particles agglomerate during the polymerization, with increased polymerization time, a uniform coating is formed around the electrospun PVDF fibers. The diameter of the electrospun PVDF fibers was measured at 3.52 ± 0.15 µm and the diameter of PPy coated PVDF fibers was 4.08 ± 0.76 µm after 18 h of polymerization. High resolution cross-sectional images of the group coated with PPy for 18 h are shown in Figure 2 (B-D). The cross-sectional images did not permit discrimination of core and shell materials of the fibers; however, the images of coated fibers showed distinct regions of base fibers (core) and distinguishable coating thickness (shell).

**FIGURE 3:**
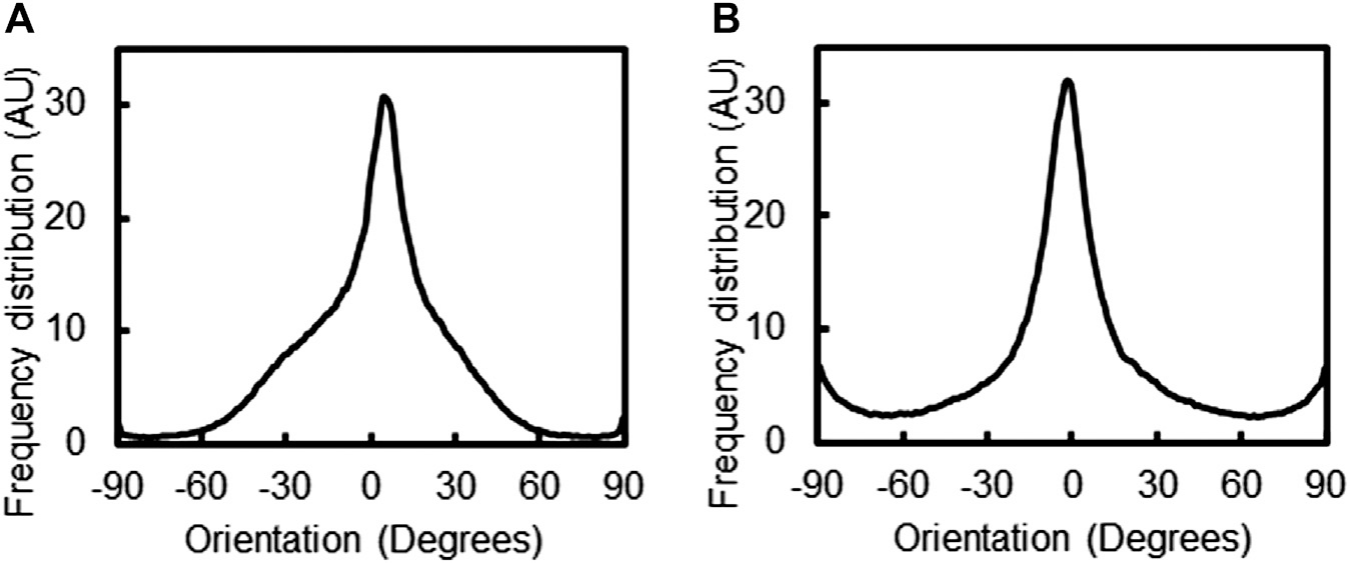
Alignment and orientation of fiber composites. The alignment of PVDF fibers **(A)** before, and **(B)** after 18 h of PPy polymerization coating was assessed by orientation distribution of fibers, measured using the OrientationJ plugin of ImageJ.

The PPy polymerization process did not impact the alignment of the PVDF fiber system, with the median orientation of the PVDF fibers found to be 4.5^o^ (z-score 3.19) and PPy coated PVDF fibers had a median orientation of −1.5^o^ (z-score 3.58), as measured by OrientationJ (Figure 3). The alignment of the PPy-coated PVDF fibers was undisturbed during the PPy polymerization process, with the debris and other PPy clusters observed in the microscopy images resulting in a minor increase in perpendicularly oriented fibers (at −90^o^ and 90^o^ respectively).

### Material Characterization of PPy-Coated PVDF-Electrospun Fibers

**FIGURE 4:**
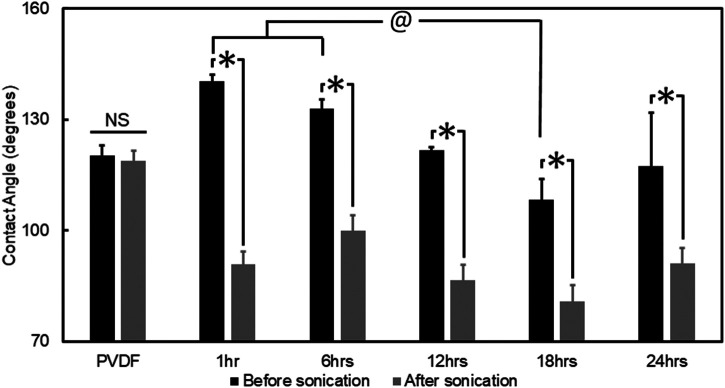
Quantified hydrophilicity of PPy coated PVDF fibers. Contact angle of PVDF fibers and PPy coated PVDF fibers were measured before and after sonication. All groups with PPy coating showed significantly lower contact angle after sonication (*, *p* < 0.001), with the PVDF fibers demonstrating no difference in contact angle before and after sonication (NS, not significant). The contact angle on the 18 h coating of PPy was significantly lower compared to the contact angle on the 1 and 6 h coatings of PPy (@, *p* < 0.005).

Hydrophilicity of PVDF fibers, and PPy coated PVDF fibers (after 1, 6, 12, 18 and 24 h of PPy polymerization) was characterized before and after sonication via sessile drop contact angle measurement. All PPy coated groups demonstrated significantly higher contact angle before sonication, compared to the contact angle on samples after sonication (*p* < 0.001, Figure 4). The highest contact angle before the sonication was observed for the sample with 1 h PPy coating (140° ± 4°) which decreased to 91° ± 4° after sonication (*p* < 0.001). All other PPy-coated groups demonstrated increased hydrophilicity after sonication and the lowest contact angle (81° ± 4°) was observed in samples coated by 18 h of PPy polymerization. The group coated by 18 h PPy polymerization had a significantly lower contact angle before sonication compared to the 1 h and 6 h PPy polymerization coatings (*p* < 0.005) and after sonication compared to the 6 h PPy polymerization coating (*p* = 0.014, Figure 4). There was no change in contact angle measured on the PVDF fibers before and after sonication (120° ± 3°, *p* = 0.811). After sonication, all PPy coated groups had significantly more hydrophilic surfaces compared to the uncoated PVDF (*p* < 0.015). These results are in line with the PPy debris observed on the PVDF fiber surfaces during the PPy polymerization process (Figure 2). The uniform coating produced after 18 h of PPy polymerization led to a reduction in contact angle, and sonication which removed PPy debris from the surface also led to a net reduction in contact angle. Since the most uniform coating, with the lowest contact angle (most hydrophilic and hence conducive for biomedical applications) was observed after 18 h of PPy polymerization on the PVDF surface, all further electrosensitive drug delivery studies were conducted using this optimized synthesis parameter. X-ray Photoelectron Spectroscopy analysis confirmed the change in chemical composition from the PVDF substrate to the PPy coated samples (Supplementary Table). The impact of polymerization time on PPy coating characteristics was best represented by the changing ratio between the percentage of F1s and N1s atoms observed from the survey spectra (Supplementary Table).

**FIGURE 5:**
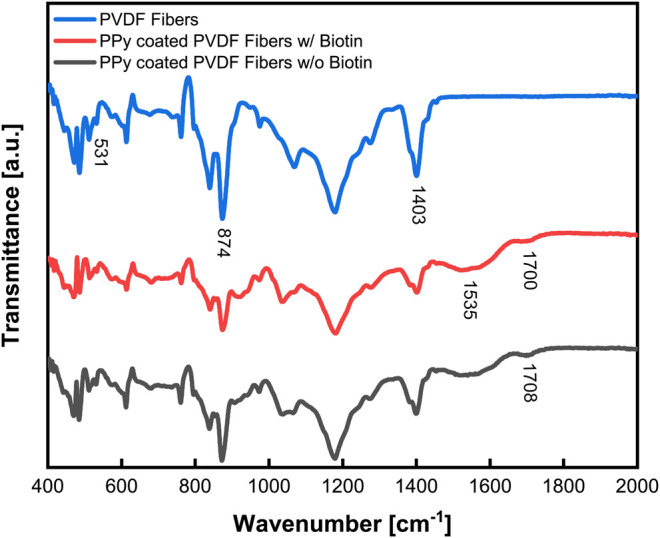
Fourier Transform Infrared Spectroscopy (FTIR) characterization of PPy coating on PVDF with biotin incorporated as a co-dopant. Spectra of PVDF electrospun fibers (top line, blue), had a high *ß* ratio of 75% indicative of piezoelectric properties, while PPy coating was demonstrated on the PVDF fibers (middle and bottom lines, red and gray) by characteristic peaks at 1,535 and 1700 cm^−1^. Evidence of biotin incorporation (red vs. gray) is seen by an overlap of peaks resulting in a shift at 1708 cm^−1^.

Attenuated Total Reflection Fourier Transform IR (ATR-FTIR) was used to characterize the piezoelectric characteristics of the electrospun PVDF, demonstrated the coating of PPy on the PVDF and established that biotin was incorporated within the PPy coating (Figure 5). Characteristic peaks observed at 531 cm^−1^ and 874 cm^−1^ correspond to the C–F bond stretching vibration of PVDF (Yee et al., 2007) while the peak at 1,403 cm^−1^ is characteristic of the CH_2_ wagging vibration (Bormashenko et al., 2004). The ratio of the β phase (more polar and hence representative of the extent of piezoelectricity) compared to the α phase was measured to be 75% using the magnitudes of the absorbance at 530 and 840 cm^−1^. The peaks at 1,535 cm^−1^ and 1700 cm^−1^ represents characteristic bonds of pyrrole which were observed in samples with PPy coating (Dams et al., 2013). Out-of-plane bending absorption was observed near 800 cm^−1^ due to the N-H bonds of PPy. Also, the characteristic wide stretch of the C=C bond was observed at 1,535 cm^−1^ and characteristics of the N-C=O bond were observed at 1700 cm^−1^, all of which confirmed the presence of the PPy coating. In addition, absorbance spectra of PPy coated samples with and without biotin added as a co-dopant clearly confirmed incorporation of biotin into the polymerized PPy coating (Figure 5). The peak observed at 1708 cm^−1^ was likely due to an overlap of the C=O bond (Bunaciu et al., 2009; Balan et al., 2012) of the carboxylic acid peak and the carbamide groups characteristic of biotin.

**FIGURE 6:**
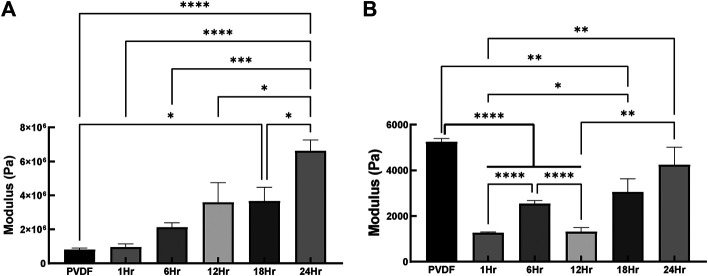
Tensile and indentation based mechanical properties **(A)** Elastic modulus based on tensile testing conducted on PVDF electrospun fibers and PVDF fibers coated with PPy for 1, 6, 12, 18, 24 h in which fibers were stretched aligned with the fiber’s orientation **(B)** Local elastic modulus of PVDF fibers and coated PVDF fibers with PPy (n = 6 samples per group, *p < 0.05, **p < 0.01, ***p < 0.001, ****p < 0.0001).

Mechanical characterization of the fibers indicated that the tensile stiffness increased in direct proportion to the duration of PPy coating, increasing significantly from 0.8 ± 0.09 MPa for PVDF to 6.6 ± 0.65 MPa for the PVDF after 24 h of PPy coating (*p* < 0.0001, Figure 6A). This is intuitively explained by the tensile stiffness of fibers being directly proportional to their diameter and the diameter increasing with coating duration. In the case of local micromechanical properties, coating the PVDF with PPy caused a significant reduction in indentation modulus (*p* < 0.0001 after 1, 6 and 12 h of coating), as seen in Figure 6B. An increase in indentation modulus was observed with increased duration of PPy coating, such that no significant difference in stiffness was observed between the bare PVDF (5.3 ± 0.14 kPa) and after 24 h of PPy coating (4.3 ± 0.76 kPa).

### Biotin Incorporation and Stability in the PPy Coating

**FIGURE 7:**
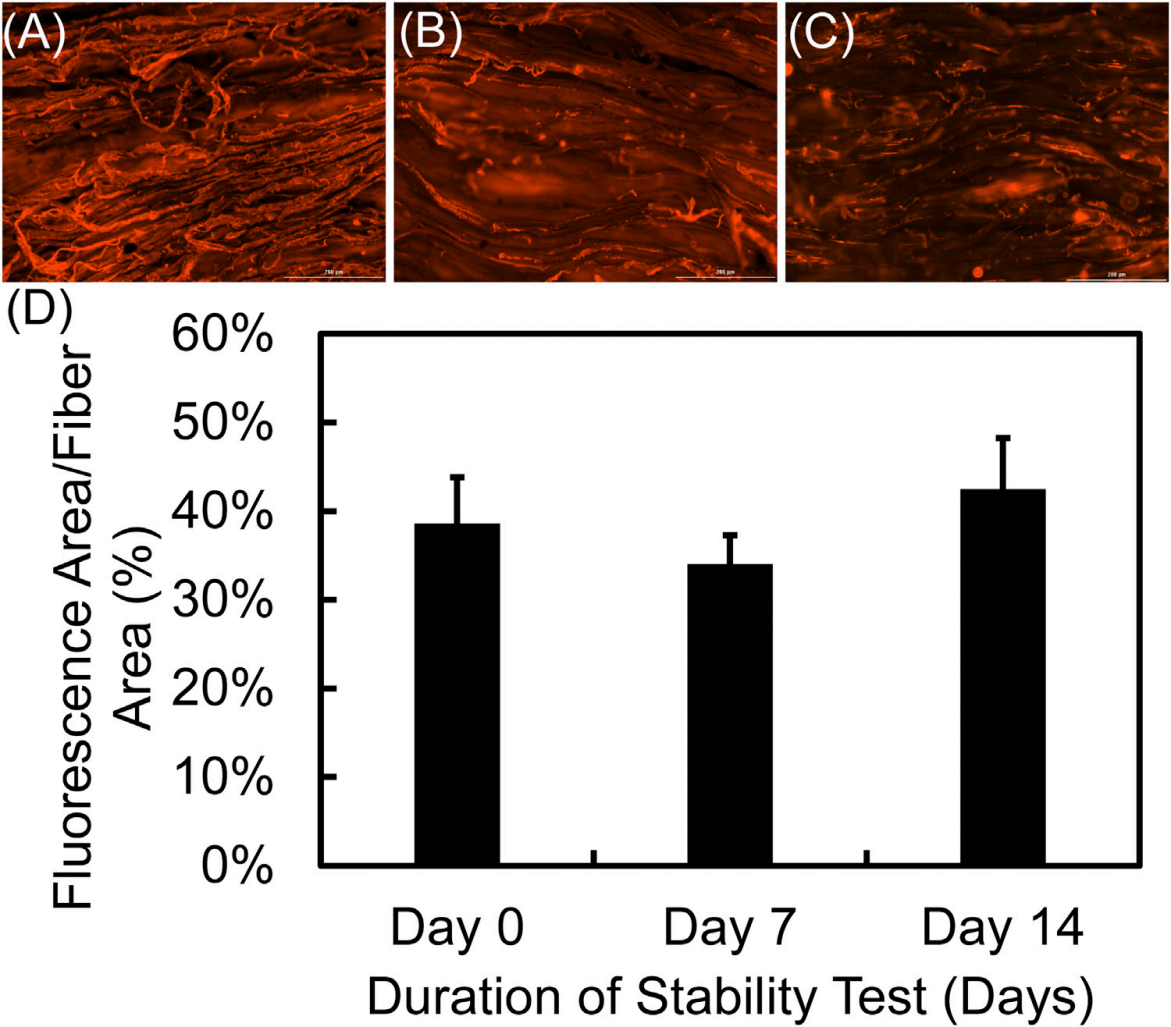
Qualitative and quantitative assessment of biotin in the PPy coating over 14 days. Fluorescent images show Alexa Fluor™ 350 conjugated streptavidin bonded to available biotin in the PPy coating after **(A)** 0 **(B)** 7, and **(C)** 14 days of incubation in phosphate buffered saline at 37 °C **(D)** The fraction of fiber area that was fluorescent was quantified over the 14 days observation period and indicated no significant change over time (*p* = 0.428).

Incorporation of biotin in the PPy coating as a dopant in the polymerization process, is essential for loading conjugated streptavidin carriers and further controlled drug delivery applications. The successful incorporation of biotin and its continued presence over 14 days as an available site for streptavidin conjugation is shown in Figure 7 by conjugation of fluorescently tagged streptavidin. Samples were stained with fluorescent-tagged streptavidin immediately upon synthesis and then after 7 days and 14 days of incubation in PBS and quantitative measurements of relative fluorescently stained area fraction of the samples indicated that there was no significant difference between the samples at different times (*p* = 0.428). Lack of significant difference between groups indicates that substantial passive release of biotin did not occur over the first 14 days.

### Conductivity and Voltage-Sensitive Release

**FIGURE 8:**
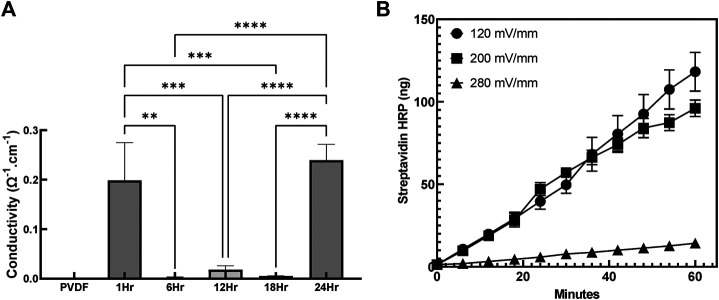
Conductivity and Electrosensitive drug release from the PPy coated PVDF **(A)** Conductivity measurements of PPy coated PVDF fibers (n = 3 samples per group, **p < 0.01, ***p < 0.001, ****p < 0.0001) **(B)** Cumulative release of Streptavidin HRP from PPy coated PVDF stimulated at 120, 200, and 280 mV/mm (at 1 Hz) electrical stimulation continuously for 60 min indicated that the streptavidin HRP released with 280 and 200 mV/mm stimulation was significantly greater than the release at 120 mV/mm (*p* < 0.05).

To evaluate the impact of coating duration on the conductivity of the sample, surface resistivity was measured using the four point probe technique and subsequently used to calculate surface conductivity (Figure 8A). The conductivity of the group coated with PPy for only 1h was relatively higher (0.19 ± 0.13Ω^−1^ cm^−1^)than groups coated with PPy for 6, 12, and 18h (0.01 ± 0.00Ω^−1^cm^−1^). No significant differences were observed in conductivity between the 6, 12 and 18h coating duration. On the other hand, the highest conductivity (0.24 ± 0.016Ω^−1^ cm^−1^) was measured in group coated with PPy for 24 h which was significantly higher that the groups coated with PPy for 6, 12, and 18 h. This result revealed the possibility of bare PVDF being present in coated groups even after 18 h of PPy coating while the coating for 24 h caused the highest conductivity potentially due to more congruent coatings, despite a significant amount of PPy debris observed in the system.

Streptavidin conjugated drug release from PPy coated PVDF fibers in response to electrical stimulation was measured by quantifying streptavidin HRP release under different stimulatory electrical regimes (120, 200, and 280 mV/mm) over 60 min (measured every 6 min), as seen in Figure 8B. The least drug quantity released was at 120 mV/mm while the drug released under both 200 and 280 mV/mm stimulation (not significantly different from each other, *p* = 0.136), each demonstrated significantly greater cumulative release (*p* < 0.001, main effect between groups). Over the first 12 min, no significant difference was observed in the total drug released between the three stimulation levels (*p* > 0.068), but from 18 min to 60 min of stimulation, significantly greater drug was released at 200 and 280 mV/mm applied electrical stimulation compared to 120 mV/mm stimulation (*p* < 0.005). The cumulative release indicated electrosensitive behavior underlying the drug release observed from the PPy coatings, with 14.3 ± 2.1, 96.1 ± 5.0, and 118.2 ± 11.7 ng of streptavidin HRP was released after 60 min of electrical stimulation at 120, 200, and 280 mV/mm respectively (Figure 8B). At 120 mV/mm there was no statistically significant difference between cumulative release after any time point (*p* > 0.08), indicating that the 200 mV/mm was the minimum stimulatory level to achieve substantial stimulus responsive release.

**FIGURE 9:**
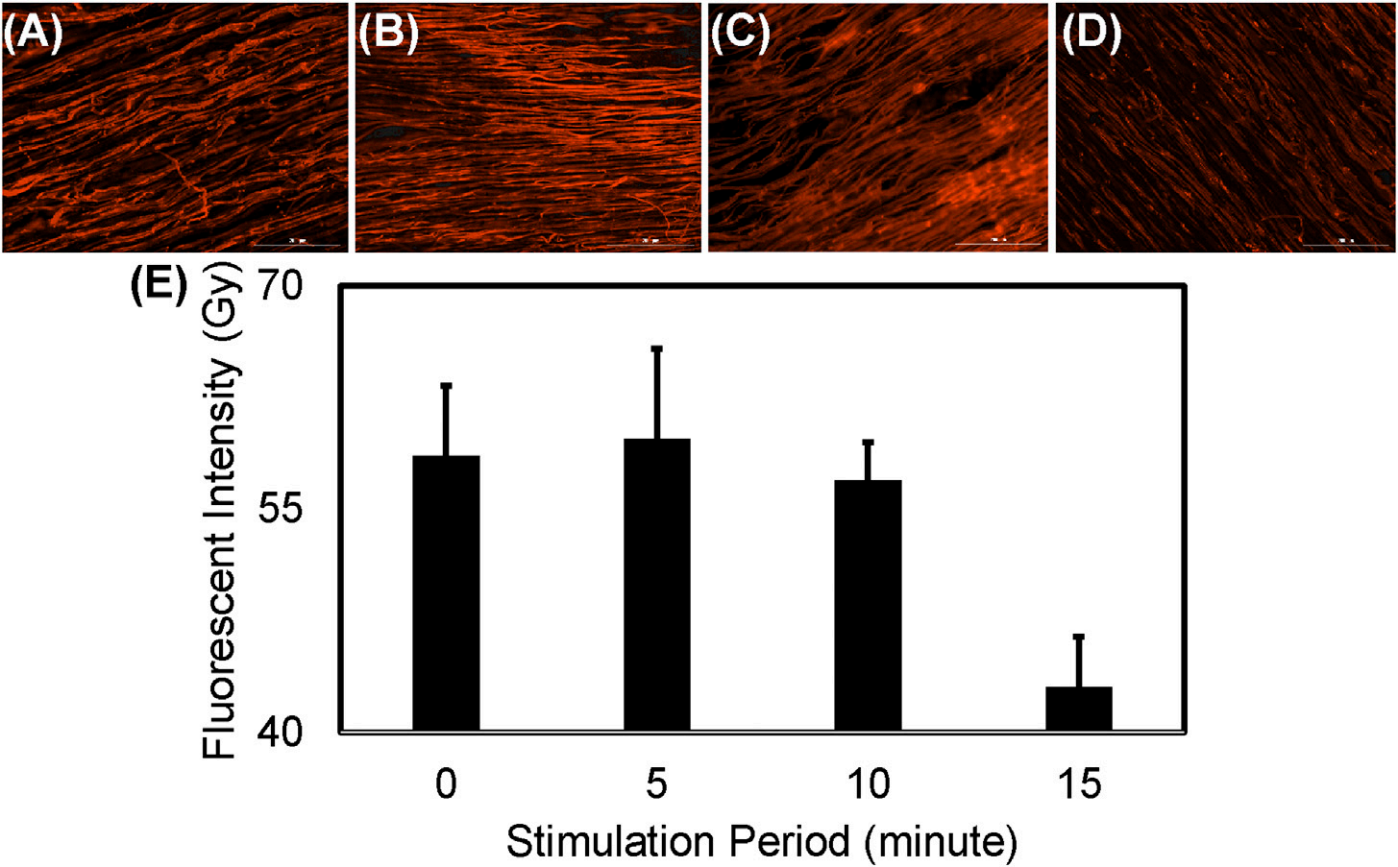
Electrical stimulus based drug delivery from PPy coated PVDF fibers. Fluorescent images show Alexa Fluor™ 350 conjugated streptavidin bound to the biotin available in the PPy coating **(A)** before and after **(B)** 5 min **(C)** 10 min or **(D)** 15 min of electrical stimulation at 200 mV/mm and 1 Hz **(E)** Quantification of fluorescence intensity demonstrates a decrease after 15 min of stimulation (not significant, *p* = 0.064).

The sustainability of the PPy-coated PVDF fibers as a drug depot (Figure 9) was demonstrated by measurement of the streptavidin Alexa Fluor™ 350 conjugate remaining in the PPy coating during 15 min of electrical stimulation (at 200 mV/mm and 1 Hz). The fluorescent intensity did not decrease during the first 5 min (*p* = 0.998) or 10 min (*p* = 0.993) of electrical stimulation and a subtle decrease was observed by 15 min of electrical stimulation (*p* = 0.09). The streptavidin remaining in the PPy coated PVDF fibers decreased slightly after 15 min of stimulation, however, the reduction in the streptavidin conjugated fluorescence in the PPy-coated PVDF fibers after 15 min of electrical stimulation was not significantly different from time as synthesized (*p* = 0.09) or 5 min of stimulation (*p* = 0.064, Figure 9). This is in accordance with the observations after continuous stimulation at 200 mV/mm and 1 Hz, no significant difference was found in cumulative drug release until after 18 min of continuous stimulation (Figure 8B).

### Growth Factor Release and Bioactivity

**FIGURE 10:**
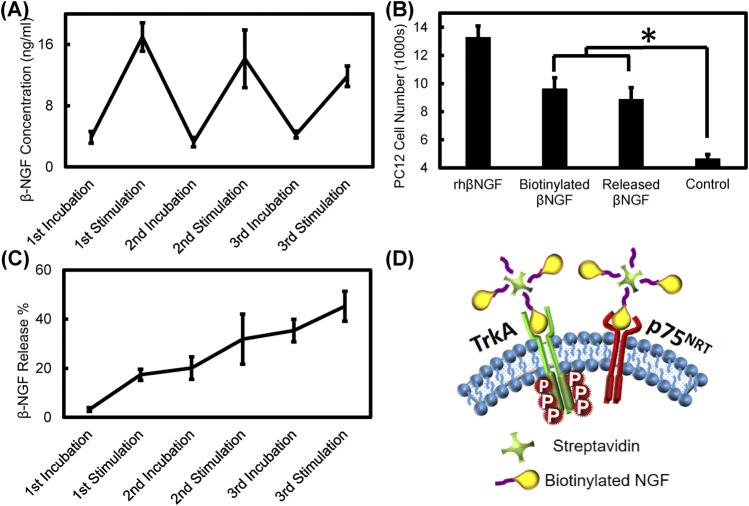
Electrical stimulation based NGF release and bioactivity assay **(A)** Streptavidin tethered biotinylated NGF release from PPy coated PVDF fibers during sequential electrical stimulation at 200 mV/mm, for 5 min and 5 min of incubation without stimulation **(B)** Bioactivity of released NGF compared to recombinant human NGF, biotinylated NGF, and control groups based on PC12 cell proliferation (*, *p* < 0.003) **(C)** Cumulative NGF released from PPy coated PVDF fibers during incubation and stimulation periods **(D)** Schematic picture of NGF receptors (TrKA and p75) in the cell membrane and the potential interaction of the drug carrier released from PPy coated PVDF fibers with the receptors. Any one of three biotinylated NGFs bound to the streptavidin can interact with the receptors.

PPy coated PVDF fibers were loaded with streptavidin conjugated with biotinylated growth factors which were then electrically stimulated at 200 mV/mm (and 1 Hz frequency). Biotinylated NGF and bFGF were separately conjugated to streptavidin, released over three intervals and the released growth factor complex was tested to ensure maintenance of bioactivity. As seen in Figure 10, the instantaneous concentration of NGF increased after each stimulation period and returned to the baseline level during incubation periods between electrical stimulation. Figure 10C shows the cumulative release of NGF, which indicates that nearly 45% of the original NGF loaded into the samples was released after electrical stimulation (for a total of 15 min). PC12 cells exposed to recombinant human NGF, biotinylated NGF, and NGF-streptavidin complex released from PPy-coated PVDF fibers at equivalent concentrations of growth factor showed that cell proliferation was significantly higher when exposed to the recombinant human NGF compared to all other treatments (*p* < 0.01), while exposure to the biotinylated NGF and released NGF resulted in significantly greater cell count than the control group with no exposure (*p* < 0.003). There was no significant difference in cell number when exposed to the biotinylated NGF compared to the released NGF (*p* = 0.891), indicating that electrical stimulation and conjugation to streptavidin did not affect activity of the NGF (Figure 10). Figure 10D illustrates the schematic picture of NGF receptor interaction with the streptavidin-biotinylated NGF complex.

**FIGURE 11:**
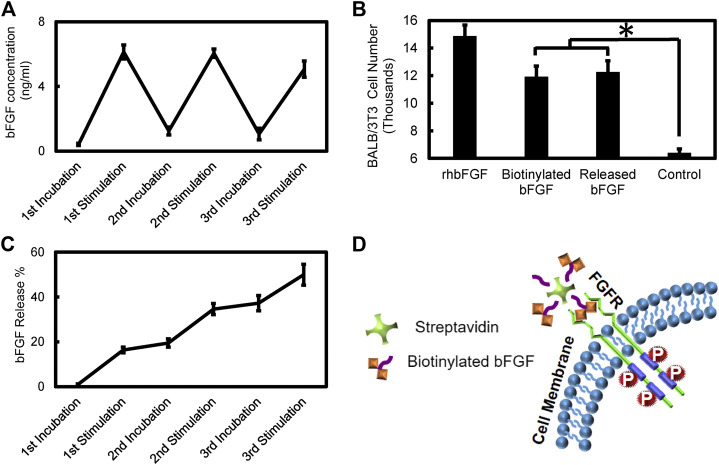
Electrical stimulation based bFGF release and bioactivity assay **(A)** bFGF release profile during sequential electrical stimulation (200 mV/mm, 1Hz, 5 min) and incubation (5 min) periods **(B)** Bioactivity of released bFGF complexes compared to recombinant human bFGF, biotinylated bFGF, and control groups with no growth factor based on BALB/c cell proliferation (*p < 0.001) **(C)** Cumulative percentage of bFGF release during sequential electrical stimulation (15 min total) and incubation periods **(D)** Schematic picture of potential FGF receptor interaction with the drug carrier released from PPy coated PVDF fibers.

Figure 11 quantifies bFGF release from the PPy coated PVDF fibers after electrical stimulation and incubation periods identical to the profile observed for NGF. bFGF release (6 ng/ml under electrical stimulation) was observed consistently during every sequence of electrical stimulation. 49% of the bFGF loaded in the PPy coating was released during the three alternating sets of 5 min stimulation followed by 5 min incubation period sequences. Bioactivity of the released bFGF based on BALB cell proliferation showed that there was no significant difference in the activity of the released bFGF and biotinylated bFGF on cell proliferation (*p* = 0.934) while both these treatments resulted in significantly higher cell proliferation compared to the control group with no growth factor exposure (*p* < 0.001). The recombinant human bFGF treatment resulted in significantly higher cell proliferation compared to all other treatments (*p* < 0.001). Figure 11D shows the schematic of the drug carrier released from PPy coated PVDF fibers and its interaction with the cell surface fibroblast growth factor receptor. As previously mentioned, the receptor interaction with (one of up to three) available growth factors bound to streptavidin.

## Discussion

Electro-conductive polymeric coatings are essential to leverage the application of electrical potential or current in biomedical applications. Poly-pyrrole (PPy) has been one of the most extensively used polymers for such applications, and the synthesis method used has mostly been electrochemical polymerization (Huang et al., 2014; Liang and Goh, 2020). While this process has been used to great effect in creating materials suitable for nerve and muscle tissue engineering *in vitro*, it is limited to creating coatings on electrodes (Liang and Goh, 2020), inherently incompatible with implantable systems and *in vivo* studies. On the other hand, *in situ* polymerization is another technique for generating PPy coatings (Tada et al., 2002; Ferenets and Harlin, 2007; Vo et al., 2012; Hammad et al., 2018; Xie et al., 2019); which can be more broadly applied in terms of substrate materials and thus has the potential for more far-reaching applications in tissue engineering and drug delivery.

PPy chain growth is virtually unlimited in electrochemical polymerization due to inherent conductivity (Ateh et al., 2006) and offers advantages in controlling the deposition of thin polymeric films, while polymer chain growth terminates and PPy particles are formed and subsequently deposited during *in situ* polymerization (Ansari, 2006; Bahraeian et al., 2013). Particulate deposition might seem like a disadvantage to controlling film thickness, but particles formed during *in situ* polymerization can then be deposited as a coating regardless of the nature of the substrate (Zhang and Manohar, 2004). In the present study, we observed that the deposition of PPy on PVDF fibers began within the first hours of polymerization. While both particulate PPy on the surface as well as increasingly cohesive coating were observed with increased PPy polymerization time (Figure 2), the observation suggests the site-selective interaction of PPy with PVDF in terms of initial nucleation site. Microfibers are known to have relatively higher surface area compared to films (Huang et al., 2008), which has a crucial impact on the quality of *in situ* polymerized PPy coating from a more efficient diffusion and migration of the monomer into the fibrous matrix (Liang et al., 2014). In addition, the PVDF fibers being prepared by electrospinning results in a higher β phase in the PVDF. The β phase, one of four phases in PVDF, has the highest spontaneous polarization, and results in the most piezoelectric properties since due to highly electronegative C-F bonds (Li et al., 2019; Mishra et al., 2019) and dipoles aligned in the same direction. The polarized surface of PVDF fibers is another potential reason for improved coating of PPy on PVDF due to the interaction of the electronegative C-F bonds in PVDF fibers with the electropositive C-N bonds (Hemant et al., 2011) in PPy. Results from the present study indicated that the nucleated PPy nanoparticles formed an increasingly cohesive coating on the individual strands of PVDF electrospun fibers over time without significant changes to the alignment of the PVDF fibers during the *in situ* polymerization process. The uniformity and cohesiveness of the coating (based on SEM micrographics) indicated that the most stable coating was formed after 18 h of polymerization, which was supported both by qualitative observation as well as quantification of surface contact angle (Figures 2 and 4).

*In situ* polymerization offers further unique advantages with regards to the choice of dopant/s included and further downstream applications leveraging the dopant/s. In the present study, the *in situ* polymerization system comprised of two dopants: sodium *p*-toluenesulfate (SPTS) and biotin (Schematic shown in Supplementary Figure). Ferric chloride was included in the *in situ* polymerization as an oxidizing agent to initiate chemical polymerization via the chlorine anions in FeCl_3_ interacting with the pyrrole rings (Brezoi, 2010; Hemant et al., 2011). SPTS is a dopant of choice for improved electrical conductivity, having been reported to result in increased stability compared to the use of hydrochloric acid (HCl) and ferric chloride (FeCl_3_) (Collier et al., 2000) as dopants resulting from a improved spatial order in PPy(Chen et al., 2003). Further, modification of the *in situ* polymerization process with biotin as an additional doping agent, enabled the loading and release of growth factors from the PPy coating for biomedical applications. PPy is a binding site for the negatively charged biotin molecules which then serve as a tunable platform for site-directed conjugate formation (Torres-Rodriguez et al., 1999; Della Pia et al., 2014; Jeon et al., 2014) and specific protein-binding for drug delivery purposes (George et al., 2006; Della Pia et al., 2014) based on widely used biotin-avidin chemistry. PPy polymerization using *in situ* polymerization has been previously developed with a variety of catalysts to prepare both coatings (including thin films) and microparticles (Liu et al., 2008; Lu et al., 2009). However, such studies have primarily focused on polymerization quality, conductivity, and the potential applications as electrical sensors/electrodes or electroactive tissue engineering scaffolds in biomedical science (Liang et al., 2014; Liang and Goh, 2020). The potential for drug loading and the use for stimulus-responsive drug release is yet to be fully investigated. In the present study, we focus on developing and characterizing the *in situ* polymerization of PPy based on a combination of dopants/catalysts which offers wide flexibility in terms of substrates. While *in situ* coating of PPy has been developed on electrospun fibers (including poly (lactic-co-glycolic acid) (Lee et al., 2009) and PVDF (Dias et al., 2014)), in the past these studies demonstrated promising results with the culture of cells and their electrical stimulation, but have not explored drug loading and delivery from such a system. In the present study, incorporating biotin as a secondary doping agent allows the functionalization of this system to allow functionalized drug/growth factor loading using biotin-avidin chemistry independent of drug chemistry, size, or electric charge. Electrical conductivity measurements in the present study are comparable or higher than previous studies of PPy coatings (Lee et al., 2009; Harjo et al., 2020). Results also demonstrated the successful incorporation of biotin, its stability and retention in the system and further, its use as a site for tethering growth factor complexes for stimulus responsive release (Figures 5, 7, and 8).

Currently, drug release systems based on PPy have two main limitations. First, the range of potential drugs is restricted due to limitations on the charge and size requirements of doping agent (Luo and Cui, 2009). In general, the rate of a dopant’s leaching from the PPy matrix decreases in dopants with higher molecular weight (Le et al., 2017), which directly affects capability for controlled drug release. In addition, the anion charge and capacity of doping agents affects the stability of the positively charged PPy (Varesano et al., 2016). In our design, by incorporation of biotin as a doping agent, we were able to test at least two separate biotinylated growth factors (bFGF and NGF), which demonstrates the potential of loading multiple, and various biotinylated growth factors within the PPy coating. The second limitation with drug delivery systems based on PPy is low drug loading efficiency, since the drug is generally incorporated in the bulk phase of PPy (Xie et al., 2017). Since *in situ* polymerization leads to shorter PPy chains and nanoparticle formation (Bahraeian et al., 2013), it is believed that the diffusion paths for the released drug are more accessible compared to the designs based on electrochemical polymerization. This phenomenon likely accounted for the ∼50% release of the loaded bFGF and NGF observed in this study over the course of 15 min of electrical stimulation (Figures 10C and 11C).

Growth factors and their delivery has been a principal tool in the tissue engineering arsenal along with the design of biomaterial architecture, and application of biophysical stimuli to induce the attachment, proliferation and tissue-specific differentiation/commitment of responsive cells. bFGF (Peng et al., 1991) and NGF (English, 2003) have been previously demonstrated to promote muscle tissue regeneration (Sakuma and Yamaguchi, 2011) as well as retain and restore poly-neuronal innervation at neuromuscular synapses (English, 2003; Sakuma and Yamaguchi, 2011). Biomaterials design is an essential aspect of neuromuscular junction (NMJ) formation since the morphology (Luo et al., 2018) surface chemistry (Das et al., 2010; Nickels and Schmidt, 2013), and conductivity (Dong et al., 2020) of biomaterials have shown to affect formation of acetylcholine receptor (AChR) clusters which are characteristic of the NMJ. Electrospun fibers such as Poly (l-lactic acid) (Zeng et al., 2013) and Poly (lactic-co-glycolic acid) (Lee et al., 2009) coated with conductive polymers (Ismail et al., 2008) such as PPy (Lee et al., 2009; Zeng et al., 2013) have been previously developed to study NMJ formation; however, the designs have mostly been limited to evidence of stable PPy coating and/or optimizing additional surface modification with immobilized growth factors (Nickels and Schmidt, 2013) [such as NGF (Gomez and Schmidt, 2007)] rather than functional release of such factors.

Nerve impulses induce depolarization of muscle fibers through released acetylcholine which inhibits AChR localization, stability, and positive signals are responsible for the accumulation of AChR (Wu et al., 2010; Lepore et al., 2019). This has been mimicked by application of external electrical stimulation in various voltage regimes and for different durations of exposure both *in vitro* and *in vivo*. As an example, exposing PC12 cells to electrical stimulation at 200 mV/mm induced neurite outgrowth and signaling (Chang et al., 2013). Additionally, applied electrical stimulation, ranging from 20 to 200 mV/mm (Hronik-Tupaj et al., 2013; Du et al., 2018; Shi et al., 2018; Hu et al., 2019), is also a crucial factor in the proliferation and successive fusion of myogenic precursor cells to form mature myofibers (Lepore et al., 2019). Neuromuscular electrical stimulation has been also widely studied *in vitro* (Di Filippo et al., 2017; Khodabukus et al., 2018) and in clinical studies (Sheffler and Chae, 2007; Wu et al., 2010; Di Filippo et al., 2017) to repair synapses, maintain motor end plates and promote functional innervation of muscle fibers (Khodabukus et al., 2018). In the present study, we used an electrical stimulation regime of 200 mV/mm which is relevant to these physiological tissue functions in addition to stimulating growth factor release from the PPy coated PVDF. Neuromuscular electrical stimulation systems allow for application of electrical stimulation of similar voltage regimes, however their combination with growth factor delivery platforms has yet to be fully explored toward successful neuromuscular junction formation. In the present study, we successfully demonstrate relevant growth factor release and continued bioactivity under the electrical regime suitable for skeletal muscle and neural cell stimulation, as well as within a range that can be potentially delivered using electrical stimulation systems. The PPy coated PVDF fiber platform also has the potential to work as a stable drug depot to release growth factors only upon applied electrical stimulation.

Skeletal muscle satellite cells and motor neurons are the central cellular constituents of neuromuscular junctions, the biological transducer of electrochemical-mechanical signals in the musculoskeletal system. In the present study, the function of the released NGF was tested on PC12 cells known to demonstrate neurite outgrowth in response and the released bFGF was tested on BALB 3T3 cells, and bFGF is known to promote survival, calcium influx and proliferation in this cell line. In addition, to form neuromuscular junctions, electrical and chemical growth factors are simultaneously required to form a synapse between motor neurons and skeletal fibers (Hultman et al., 1983; Paillard, 2008). Since the PPy-PVDF system developed in this study comprises of a piezoelectric substrate (PVDF) combined with a conducting polymer (PPy) capable of drug delivery, it offers multiple advantages in both the transduction of electrical and mechanical signals as well as coupled growth factor delivery, thus replicating roles similar to those of the neuromuscular junction. Electrical stimulation has shown to play a critical role in the production of signaling and survival factors such as acetylcholine (Mitchell, 1963; Lozano et al., 2016) and glial cell line-derived neurotrophic factor (Vianney et al., 2014). The functionality of the neuromuscular junction is correlated with the release of these growth factors in a timely manner; therefore, the scaffolds designed for muscle regeneration and NMJ studies require smart and stimulus-responsive systems to deliver essential growth factors.

## Conclusion

In this study, a novel methodology is developed to generate polypyrrole (PPy, a conductive polymer) coatings on polyvinylidene fluoride (PVDF, a piezoelectric polymer) electrospun fibers. The composite material can then be used as a facile, electrosensitive drug delivery platform for the controlled delivery of various growth factors. A stable PPy coating is achieved around PVDF electrospun fibers using an optimized *in situ* polymerization process and incorporating biotin as a co-dopant within the polymerization step, which also serves as the locus for drug-complex loading. Voltage controlled electrosensitive drug delivery of different growth factors (NGF and bFGF) was achieved, and the bioactivity of these growth factors after release was demonstrated, paving the way for potential biomedical applications ranging from soft polymeric electrodes to neuromuscular junction tissue engineering.

## Data Availability Statement

The original contributions presented in the study are included in the article/Supplementary Material, further inquiries can be directed to the corresponding author.

## Author Contributions

SM contributed to the design and writing of the present study, and conducted all experiments and data analyses. RB and JO were advisors for the design of the study and experiments as well as contributors in the writing and editing of the manuscript. TG led the design endeavor and provided oversight of all experiments, analyses and reporting of the data; and contributed to the writing and editing of the manuscript.

## Funding

This research was supported in part by the National Science Foundation CAREER Award (CBET#1847103) and Jacobson Distinguished Professorship to Teja Guda, funds from the Lutcher Brown Endowment to Rena Bizios, funds from the USAA Foundation Endowment to Joo Ong and funding from the San Antonio Life Science Institute, the UTSA Brain Health Consortium, and the UTSA Graduate School to Solaleh Miar.
