# Supplementary material for: Electrically Stimulated Tunable Drug Delivery From Polypyrrole-Coated Polyvinylidene Fluoride
Source: Front Chem. 2021 Feb 5;9:599631. doi: 10.3389/fchem.2021.599631 (PMC7892451; doi:10.3389/fchem.2021.599631)
Supplement: Supplementary file 1 [file table1.docx]

**Table 1**. Elemental composition of PVDF and PPy coated (for various durations from 1 hour to 24 hours) PVDF aligned electrospun fibers, determined using XPS analysis.

| Sample | **PVDF** | **1hr** | **6hrs** | **12hrs** | **18hrs** | **24hrs** |
| --- | --- | --- | --- | --- | --- | --- |
| **F1s %** | 99.67 | 83.24 | 50.43 | 25.54 | 22.72 | 19.9 |
| **N1s %** | 0.33 | 16.76 | 49.57 | 74.46 | 77.28 | 80.1 |
