# Supplementary material for: Electrically Stimulated Tunable Drug Delivery From Polypyrrole-Coated Polyvinylidene Fluoride
Source: Front Chem. 2021 Feb 5;9:599631. doi: 10.3389/fchem.2021.599631 (PMC7892451; doi:10.3389/fchem.2021.599631)
Supplement: Supplementary file 2 [file table2.docx]

**Contact Angle**

**Two Way Analysis of Variance** Saturday, June 20, 2020, 11:51:51 PM

**Data source:** Data 1 in Notebook1

General Linear Model

Dependent Variable: Contact Angle

**Normality Test (Shapiro-Wilk):**  Passed (P = 0.161)

**Equal Variance Test (Brown-Forsythe):** Passed (P = 0.582)

**Source of Variation DF SS MS F P**

Group 5 3173.193 634.639 11.214 <0.001

Sonication 1 8548.672 8548.672 151.055 <0.001

Group x Sonication 5 2142.307 428.461 7.571 <0.001

Residual 30 1697.792 56.593

Total 41 15794.181 385.224

Main effects cannot be properly interpreted if significant interaction is determined. This is because the size of a factor's effect depends upon the level of the other factor.

The effect of different levels of Group depends on what level of Sonication is present. There is a statistically significant interaction between Group and Sonication. (P = <0.001)

Power of performed test with alpha = 0.0500: for Group : 1.000

Power of performed test with alpha = 0.0500: for Sonication : 1.000

Power of performed test with alpha = 0.0500: for Group x Sonication : 0.991

Least square means for Group :

**Group Mean SEM**

PVDF 119.542 2.873

1hr 115.542 2.873

6hr 116.446 2.873

12hr 104.196 2.873

18hr 94.608 2.873

24hr 104.179 2.873

Least square means for Sonication :

**Group Mean SEM**

Before 123.500 1.773

After 94.671 1.536

Least square means for Group x Sonication :

**Group Mean SEM**

PVDF x Before 120.233 4.343

PVDF x After 118.850 3.761

1hr x Before 140.333 4.343

1hr x After 90.750 3.761

6hr x Before 132.967 4.343

6hr x After 99.925 3.761

12hr x Before 121.767 4.343

12hr x After 86.625 3.761

18hr x Before 108.367 4.343

18hr x After 80.850 3.761

24hr x Before 117.333 4.343

24hr x After 91.025 3.761

All Pairwise Multiple Comparison Procedures (Tukey Test):

Comparisons for factor: **Group**

**Comparison Diff of Means p q P P<0.050**

PVDF vs. 18hr 24.933 6 8.679 <0.001 Yes

PVDF vs. 24hr 15.362 6 5.348 0.008 Yes

PVDF vs. 12hr 15.346 6 5.342 0.008 Yes

PVDF vs. 1hr 4.000 6 1.392 0.919 No

PVDF vs. 6hr 3.096 6 1.078 0.972 Do Not Test

6hr vs. 18hr 21.837 6 7.601 <0.001 Yes

6hr vs. 24hr 12.267 6 4.270 0.053 No

6hr vs. 12hr 12.250 6 4.264 0.053 Do Not Test

6hr vs. 1hr 0.904 6 0.315 1.000 Do Not Test

1hr vs. 18hr 20.933 6 7.287 <0.001 Yes

1hr vs. 24hr 11.362 6 3.955 0.086 Do Not Test

1hr vs. 12hr 11.346 6 3.949 0.087 Do Not Test

12hr vs. 18hr 9.587 6 3.337 0.203 No

12hr vs. 24hr 0.0167 6 0.00580 1.000 Do Not Test

24hr vs. 18hr 9.571 6 3.331 0.204 Do Not Test

Comparisons for factor: **Sonication**

**Comparison Diff of Means p q P P<0.050**

Before vs. After 28.829 2 17.381 <0.001 Yes

Comparisons for factor: **Sonication within PVDF**

**Comparison Diff of Means p q P P<0.050**

Before vs. After 1.383 2 0.340 0.811 No

Comparisons for factor: **Sonication within 1hr**

**Comparison Diff of Means p q P P<0.050**

Before vs. After 49.583 2 12.204 <0.001 Yes

Comparisons for factor: **Sonication within 6hr**

**Comparison Diff of Means p q P P<0.050**

Before vs. After 33.042 2 8.133 <0.001 Yes

Comparisons for factor: **Sonication within 12hr**

**Comparison Diff of Means p q P P<0.050**

Before vs. After 35.142 2 8.650 <0.001 Yes

Comparisons for factor: **Sonication within 18hr**

**Comparison Diff of Means p q P P<0.050**

Before vs. After 27.517 2 6.773 <0.001 Yes

Comparisons for factor: **Sonication within 24hr**

**Comparison Diff of Means p q P P<0.050**

Before vs. After 26.308 2 6.475 <0.001 Yes

Comparisons for factor: **Group within Before**

**Comparison Diff of Means p q P P<0.050**

1hr vs. 18hr 31.967 6 7.360 <0.001 Yes

1hr vs. 24hr 23.000 6 5.295 0.009 Yes

1hr vs. PVDF 20.100 6 4.628 0.029 Yes

1hr vs. 12hr 18.567 6 4.275 0.052 No

1hr vs. 6hr 7.367 6 1.696 0.834 Do Not Test

6hr vs. 18hr 24.600 6 5.664 0.005 Yes

6hr vs. 24hr 15.633 6 3.599 0.143 No

6hr vs. PVDF 12.733 6 2.932 0.328 Do Not Test

6hr vs. 12hr 11.200 6 2.579 0.467 Do Not Test

12hr vs. 18hr 13.400 6 3.085 0.276 No

12hr vs. 24hr 4.433 6 1.021 0.978 Do Not Test

12hr vs. PVDF 1.533 6 0.353 1.000 Do Not Test

PVDF vs. 18hr 11.867 6 2.732 0.404 Do Not Test

PVDF vs. 24hr 2.900 6 0.668 0.997 Do Not Test

24hr vs. 18hr 8.967 6 2.064 0.691 Do Not Test

Comparisons for factor: **Group within After**

**Comparison Diff of Means p q P P<0.050**

PVDF vs. 18hr 38.000 6 10.103 <0.001 Yes

PVDF vs. 12hr 32.225 6 8.567 <0.001 Yes

PVDF vs. 1hr 28.100 6 7.471 <0.001 Yes

PVDF vs. 24hr 27.825 6 7.397 <0.001 Yes

PVDF vs. 6hr 18.925 6 5.031 0.015 Yes

6hr vs. 18hr 19.075 6 5.071 0.014 Yes

6hr vs. 12hr 13.300 6 3.536 0.156 No

6hr vs. 1hr 9.175 6 2.439 0.527 Do Not Test

6hr vs. 24hr 8.900 6 2.366 0.559 Do Not Test

24hr vs. 18hr 10.175 6 2.705 0.414 No

24hr vs. 12hr 4.400 6 1.170 0.960 Do Not Test

24hr vs. 1hr 0.275 6 0.0731 1.000 Do Not Test

1hr vs. 18hr 9.900 6 2.632 0.444 Do Not Test

1hr vs. 12hr 4.125 6 1.097 0.970 Do Not Test

12hr vs. 18hr 5.775 6 1.535 0.883 Do Not Test

A result of "Do Not Test" occurs for a comparison when no significant difference is found between two means that enclose that comparison. For example, if you had four means sorted in order, and found no difference between means 4 vs. 2, then you would not test 4 vs. 3 and 3 vs. 2, but still test 4 vs. 1 and 3 vs. 1 (4 vs. 3 and 3 vs. 2 are enclosed by 4 vs. 2: 4 3 2 1). Note that not testing the enclosed means is a procedural rule, and a result of Do Not Test should be treated as if there is no significant difference between the means, even though one may appear to exist.

**Biotin Stability**

**One Way Analysis of Variance** Tuesday, July 21, 2020, 1:53:54 AM

**Data source:** Data 1 in Notebook1

Dependent Variable: Fluorescence Biotin Stability

**Normality Test (Shapiro-Wilk):**  Passed (P = 0.260)

**Equal Variance Test (Brown-Forsythe):** Passed (P = 0.055)

**Group Name N Missing Mean Std Dev SEM**

Day 0 6 0 0.386 0.117 0.0479

Day 7 6 0 0.340 0.0725 0.0296

Day 14 6 0 0.425 0.129 0.0528

**Source of Variation DF SS MS F P**

Between Groups 2 0.0214 0.0107 0.898 0.428

Residual 15 0.179 0.0119

Total 17 0.200

The differences in the mean values among the treatment groups are not great enough to exclude the possibility that the difference is due to random sampling variability; there is not a statistically significant difference (P = 0.428).

Power of performed test with alpha = 0.050: --

**Voltage Based Drug Release**

**Two Way Analysis of Variance** Tuesday, July 21, 2020, 2:23:06 AM

**Data source:** Data 1 in Notebook2

Balanced Design

Dependent Variable: Release

**Normality Test (Shapiro-Wilk):**  Failed (P < 0.050)

**Equal Variance Test (Brown-Forsythe):** Failed (P < 0.050)

**Source of Variation DF SS MS F P**

Voltage 2 63896.148 31948.074 294.816 <0.001

Time 10 79450.901 7945.090 73.317 <0.001

Voltage x Time 20 29138.759 1456.938 13.445 <0.001

Residual 99 10728.247 108.366

Total 131 183214.055 1398.581

Main effects cannot be properly interpreted if significant interaction is determined. This is because the size of a factor's effect depends upon the level of the other factor.

The effect of different levels of Voltage depends on what level of Time is present. There is a statistically significant interaction between Voltage and Time. (P = <0.001)

Power of performed test with alpha = 0.0500: for Voltage : 1.000

Power of performed test with alpha = 0.0500: for Time : 1.000

Power of performed test with alpha = 0.0500: for Voltage x Time : 1.000

Least square means for Voltage :

**Group Mean**

7V 56.101

5V 51.822

3V 7.437

Std Err of LS Mean = 1.569

Least square means for Time :

**Group Mean**

0.000 1.298

6.000 7.466

12.000 13.959

18.000 20.668

24.000 30.839

30.000 38.198

36.000 47.720

42.000 54.862

48.000 62.653

54.000 69.145

60.000 76.179

Std Err of LS Mean = 3.005

Least square means for Voltage x Time :

**Group Mean**

7V x 0.000 1.298

7V x 6.000 10.713

7V x 12.000 19.802

7V x 18.000 29.216

7V x 24.000 39.604

7V x 30.000 49.668

7V x 36.000 68.171

7V x 42.000 80.507

7V x 48.000 92.518

7V x 54.000 107.451

7V x 60.000 118.164

5V x 0.000 1.298

5V x 6.000 9.739

5V x 12.000 18.828

5V x 18.000 28.242

5V x 24.000 47.071

5V x 30.000 57.134

5V x 36.000 66.224

5V x 42.000 74.014

5V x 48.000 84.078

5V x 54.000 87.324

5V x 60.000 96.089

3V x 0.000 1.298

3V x 6.000 1.948

3V x 12.000 3.246

3V x 18.000 4.545

3V x 24.000 5.843

3V x 30.000 7.791

3V x 36.000 8.765

3V x 42.000 10.063

3V x 48.000 11.362

3V x 54.000 12.660

3V x 60.000 14.284

Std Err of LS Mean = 5.205

All Pairwise Multiple Comparison Procedures (Tukey Test):

Comparisons for factor: **Voltage**

**Comparison Diff of Means p q P P<0.050**

7V vs. 3V 48.664 3 31.009 <0.001 Yes

7V vs. 5V 4.279 3 2.727 0.136 No

5V vs. 3V 44.385 3 28.282 <0.001 Yes

Comparisons for factor: **Time**

**Comparison Diff of Means p q P P<0.050**

60.000 vs. 0.000 74.880 11 24.918 <0.001 Yes

60.000 vs. 6.000 68.712 11 22.865 <0.001 Yes

60.000 vs. 12.000 62.220 11 20.705 <0.001 Yes

60.000 vs. 18.000 55.511 11 18.472 <0.001 Yes

60.000 vs. 24.000 45.339 11 15.088 <0.001 Yes

60.000 vs. 30.000 37.981 11 12.639 <0.001 Yes

60.000 vs. 36.000 28.459 11 9.470 <0.001 Yes

60.000 vs. 42.000 21.317 11 7.094 <0.001 Yes

60.000 vs. 48.000 13.526 11 4.501 0.068 No

60.000 vs. 54.000 7.034 11 2.341 0.854 Do Not Test

54.000 vs. 0.000 67.847 11 22.577 <0.001 Yes

54.000 vs. 6.000 61.679 11 20.525 <0.001 Yes

54.000 vs. 12.000 55.186 11 18.364 <0.001 Yes

54.000 vs. 18.000 48.477 11 16.132 <0.001 Yes

54.000 vs. 24.000 38.306 11 12.747 <0.001 Yes

54.000 vs. 30.000 30.948 11 10.298 <0.001 Yes

54.000 vs. 36.000 21.425 11 7.130 <0.001 Yes

54.000 vs. 42.000 14.284 11 4.753 0.042 Yes

54.000 vs. 48.000 6.493 11 2.161 0.907 Do Not Test

48.000 vs. 0.000 61.354 11 20.417 <0.001 Yes

48.000 vs. 6.000 55.186 11 18.364 <0.001 Yes

48.000 vs. 12.000 48.694 11 16.204 <0.001 Yes

48.000 vs. 18.000 41.985 11 13.971 <0.001 Yes

48.000 vs. 24.000 31.813 11 10.586 <0.001 Yes

48.000 vs. 30.000 24.455 11 8.138 <0.001 Yes

48.000 vs. 36.000 14.933 11 4.969 0.027 Yes

48.000 vs. 42.000 7.791 11 2.593 0.758 No

42.000 vs. 0.000 53.563 11 17.824 <0.001 Yes

42.000 vs. 6.000 47.395 11 15.772 <0.001 Yes

42.000 vs. 12.000 40.903 11 13.611 <0.001 Yes

42.000 vs. 18.000 34.194 11 11.379 <0.001 Yes

42.000 vs. 24.000 24.022 11 7.994 <0.001 Yes

42.000 vs. 30.000 16.664 11 5.545 0.007 Yes

42.000 vs. 36.000 7.142 11 2.377 0.842 No

36.000 vs. 0.000 46.421 11 15.448 <0.001 Yes

36.000 vs. 6.000 40.253 11 13.395 <0.001 Yes

36.000 vs. 12.000 33.761 11 11.235 <0.001 Yes

36.000 vs. 18.000 27.052 11 9.002 <0.001 Yes

36.000 vs. 24.000 16.880 11 5.617 0.006 Yes

36.000 vs. 30.000 9.522 11 3.169 0.482 No

30.000 vs. 0.000 36.899 11 12.279 <0.001 Yes

30.000 vs. 6.000 30.731 11 10.226 <0.001 Yes

30.000 vs. 12.000 24.239 11 8.066 <0.001 Yes

30.000 vs. 18.000 17.530 11 5.833 0.004 Yes

30.000 vs. 24.000 7.358 11 2.449 0.816 No

24.000 vs. 0.000 29.541 11 9.830 <0.001 Yes

24.000 vs. 6.000 23.373 11 7.778 <0.001 Yes

24.000 vs. 12.000 16.880 11 5.617 0.006 Yes

24.000 vs. 18.000 10.172 11 3.385 0.382 No

18.000 vs. 0.000 19.369 11 6.446 <0.001 Yes

18.000 vs. 6.000 13.201 11 4.393 0.083 No

18.000 vs. 12.000 6.709 11 2.233 0.888 Do Not Test

12.000 vs. 0.000 12.660 11 4.213 0.115 No

12.000 vs. 6.000 6.493 11 2.161 0.907 Do Not Test

6.000 vs. 0.000 6.168 11 2.052 0.932 Do Not Test

Comparisons for factor: **Time within 7V**

**Comparison Diff of Means p q P P<0.050**

60.000 vs. 0.000 116.865 11 22.453 <0.001 Yes

60.000 vs. 6.000 107.451 11 20.644 <0.001 Yes

60.000 vs. 12.000 98.361 11 18.898 <0.001 Yes

60.000 vs. 18.000 88.947 11 17.089 <0.001 Yes

60.000 vs. 24.000 78.559 11 15.093 <0.001 Yes

60.000 vs. 30.000 68.496 11 13.160 <0.001 Yes

60.000 vs. 36.000 49.992 11 9.605 <0.001 Yes

60.000 vs. 42.000 37.657 11 7.235 <0.001 Yes

60.000 vs. 48.000 25.645 11 4.927 0.029 Yes

60.000 vs. 54.000 10.713 11 2.058 0.931 No

54.000 vs. 0.000 106.152 11 20.394 <0.001 Yes

54.000 vs. 6.000 96.738 11 18.586 <0.001 Yes

54.000 vs. 12.000 87.649 11 16.839 <0.001 Yes

54.000 vs. 18.000 78.235 11 15.031 <0.001 Yes

54.000 vs. 24.000 67.847 11 13.035 <0.001 Yes

54.000 vs. 30.000 57.783 11 11.102 <0.001 Yes

54.000 vs. 36.000 39.280 11 7.547 <0.001 Yes

54.000 vs. 42.000 26.944 11 5.177 0.017 Yes

54.000 vs. 48.000 14.933 11 2.869 0.630 No

48.000 vs. 0.000 91.220 11 17.526 <0.001 Yes

48.000 vs. 6.000 81.805 11 15.717 <0.001 Yes

48.000 vs. 12.000 72.716 11 13.971 <0.001 Yes

48.000 vs. 18.000 63.302 11 12.162 <0.001 Yes

48.000 vs. 24.000 52.914 11 10.166 <0.001 Yes

48.000 vs. 30.000 42.850 11 8.233 <0.001 Yes

48.000 vs. 36.000 24.347 11 4.678 0.048 Yes

48.000 vs. 42.000 12.011 11 2.308 0.865 No

42.000 vs. 0.000 79.209 11 15.218 <0.001 Yes

42.000 vs. 6.000 69.794 11 13.409 <0.001 Yes

42.000 vs. 12.000 60.705 11 11.663 <0.001 Yes

42.000 vs. 18.000 51.291 11 9.854 <0.001 Yes

42.000 vs. 24.000 40.903 11 7.858 <0.001 Yes

42.000 vs. 30.000 30.839 11 5.925 0.003 Yes

42.000 vs. 36.000 12.336 11 2.370 0.844 No

36.000 vs. 0.000 66.873 11 12.848 <0.001 Yes

36.000 vs. 6.000 57.459 11 11.039 <0.001 Yes

36.000 vs. 12.000 48.369 11 9.293 <0.001 Yes

36.000 vs. 18.000 38.955 11 7.484 <0.001 Yes

36.000 vs. 24.000 28.567 11 5.488 0.008 Yes

36.000 vs. 30.000 18.504 11 3.555 0.310 No

30.000 vs. 0.000 48.369 11 9.293 <0.001 Yes

30.000 vs. 6.000 38.955 11 7.484 <0.001 Yes

30.000 vs. 12.000 29.866 11 5.738 0.005 Yes

30.000 vs. 18.000 20.451 11 3.929 0.182 No

30.000 vs. 24.000 10.063 11 1.933 0.953 Do Not Test

24.000 vs. 0.000 38.306 11 7.359 <0.001 Yes

24.000 vs. 6.000 28.892 11 5.551 0.007 Yes

24.000 vs. 12.000 19.802 11 3.804 0.220 No

24.000 vs. 18.000 10.388 11 1.996 0.943 Do Not Test

18.000 vs. 0.000 27.918 11 5.364 0.011 Yes

18.000 vs. 6.000 18.504 11 3.555 0.310 No

18.000 vs. 12.000 9.414 11 1.809 0.970 Do Not Test

12.000 vs. 0.000 18.504 11 3.555 0.310 No

12.000 vs. 6.000 9.090 11 1.746 0.977 Do Not Test

6.000 vs. 0.000 9.414 11 1.809 0.970 Do Not Test

Comparisons for factor: **Time within 5V**

**Comparison Diff of Means p q P P<0.050**

60.000 vs. 0.000 94.791 11 18.212 <0.001 Yes

60.000 vs. 6.000 86.350 11 16.590 <0.001 Yes

60.000 vs. 12.000 77.261 11 14.844 <0.001 Yes

60.000 vs. 18.000 67.847 11 13.035 <0.001 Yes

60.000 vs. 24.000 49.018 11 9.418 <0.001 Yes

60.000 vs. 30.000 38.955 11 7.484 <0.001 Yes

60.000 vs. 36.000 29.866 11 5.738 0.005 Yes

60.000 vs. 42.000 22.075 11 4.241 0.109 No

60.000 vs. 48.000 12.011 11 2.308 0.865 Do Not Test

60.000 vs. 54.000 8.765 11 1.684 0.982 Do Not Test

54.000 vs. 0.000 86.026 11 16.528 <0.001 Yes

54.000 vs. 6.000 77.585 11 14.906 <0.001 Yes

54.000 vs. 12.000 68.496 11 13.160 <0.001 Yes

54.000 vs. 18.000 59.082 11 11.351 <0.001 Yes

54.000 vs. 24.000 40.253 11 7.734 <0.001 Yes

54.000 vs. 30.000 30.190 11 5.800 0.004 Yes

54.000 vs. 36.000 21.101 11 4.054 0.149 No

54.000 vs. 42.000 13.310 11 2.557 0.773 Do Not Test

54.000 vs. 48.000 3.246 11 0.624 1.000 Do Not Test

48.000 vs. 0.000 82.779 11 15.904 <0.001 Yes

48.000 vs. 6.000 74.339 11 14.282 <0.001 Yes

48.000 vs. 12.000 65.250 11 12.536 <0.001 Yes

48.000 vs. 18.000 55.836 11 10.727 <0.001 Yes

48.000 vs. 24.000 37.007 11 7.110 <0.001 Yes

48.000 vs. 30.000 26.944 11 5.177 0.017 Yes

48.000 vs. 36.000 17.854 11 3.430 0.362 Do Not Test

48.000 vs. 42.000 10.063 11 1.933 0.953 Do Not Test

42.000 vs. 0.000 72.716 11 13.971 <0.001 Yes

42.000 vs. 6.000 64.276 11 12.349 <0.001 Yes

42.000 vs. 12.000 55.186 11 10.603 <0.001 Yes

42.000 vs. 18.000 45.772 11 8.794 <0.001 Yes

42.000 vs. 24.000 26.944 11 5.177 0.017 Yes

42.000 vs. 30.000 16.880 11 3.243 0.447 No

42.000 vs. 36.000 7.791 11 1.497 0.993 Do Not Test

36.000 vs. 0.000 64.925 11 12.474 <0.001 Yes

36.000 vs. 6.000 56.485 11 10.852 <0.001 Yes

36.000 vs. 12.000 47.395 11 9.106 <0.001 Yes

36.000 vs. 18.000 37.981 11 7.297 <0.001 Yes

36.000 vs. 24.000 19.153 11 3.680 0.262 No

36.000 vs. 30.000 9.090 11 1.746 0.977 Do Not Test

30.000 vs. 0.000 55.836 11 10.727 <0.001 Yes

30.000 vs. 6.000 47.395 11 9.106 <0.001 Yes

30.000 vs. 12.000 38.306 11 7.359 <0.001 Yes

30.000 vs. 18.000 28.892 11 5.551 0.007 Yes

30.000 vs. 24.000 10.063 11 1.933 0.953 Do Not Test

24.000 vs. 0.000 45.772 11 8.794 <0.001 Yes

24.000 vs. 6.000 37.332 11 7.172 <0.001 Yes

24.000 vs. 12.000 28.242 11 5.426 0.010 Yes

24.000 vs. 18.000 18.828 11 3.617 0.285 No

18.000 vs. 0.000 26.944 11 5.177 0.017 Yes

18.000 vs. 6.000 18.504 11 3.555 0.310 No

18.000 vs. 12.000 9.414 11 1.809 0.970 Do Not Test

12.000 vs. 0.000 17.530 11 3.368 0.389 No

12.000 vs. 6.000 9.090 11 1.746 0.977 Do Not Test

6.000 vs. 0.000 8.440 11 1.622 0.987 Do Not Test

Comparisons for factor: **Time within 3V**

**Comparison Diff of Means p q P P<0.050**

60.000 vs. 0.000 12.985 11 2.495 0.798 No

60.000 vs. 6.000 12.336 11 2.370 0.844 Do Not Test

60.000 vs. 12.000 11.037 11 2.121 0.917 Do Not Test

60.000 vs. 18.000 9.739 11 1.871 0.963 Do Not Test

60.000 vs. 24.000 8.440 11 1.622 0.987 Do Not Test

60.000 vs. 30.000 6.492 11 1.247 0.998 Do Not Test

60.000 vs. 36.000 5.519 11 1.060 1.000 Do Not Test

60.000 vs. 42.000 4.220 11 0.811 1.000 Do Not Test

60.000 vs. 48.000 2.922 11 0.561 1.000 Do Not Test

60.000 vs. 54.000 1.623 11 0.312 1.000 Do Not Test

54.000 vs. 0.000 11.362 11 2.183 0.901 Do Not Test

54.000 vs. 6.000 10.713 11 2.058 0.931 Do Not Test

54.000 vs. 12.000 9.414 11 1.809 0.970 Do Not Test

54.000 vs. 18.000 8.116 11 1.559 0.990 Do Not Test

54.000 vs. 24.000 6.817 11 1.310 0.998 Do Not Test

54.000 vs. 30.000 4.869 11 0.936 1.000 Do Not Test

54.000 vs. 36.000 3.895 11 0.748 1.000 Do Not Test

54.000 vs. 42.000 2.597 11 0.499 1.000 Do Not Test

54.000 vs. 48.000 1.298 11 0.249 1.000 Do Not Test

48.000 vs. 0.000 10.063 11 1.933 0.953 Do Not Test

48.000 vs. 6.000 9.414 11 1.809 0.970 Do Not Test

48.000 vs. 12.000 8.116 11 1.559 0.990 Do Not Test

48.000 vs. 18.000 6.817 11 1.310 0.998 Do Not Test

48.000 vs. 24.000 5.519 11 1.060 1.000 Do Not Test

48.000 vs. 30.000 3.571 11 0.686 1.000 Do Not Test

48.000 vs. 36.000 2.597 11 0.499 1.000 Do Not Test

48.000 vs. 42.000 1.299 11 0.249 1.000 Do Not Test

42.000 vs. 0.000 8.765 11 1.684 0.982 Do Not Test

42.000 vs. 6.000 8.116 11 1.559 0.990 Do Not Test

42.000 vs. 12.000 6.817 11 1.310 0.998 Do Not Test

42.000 vs. 18.000 5.519 11 1.060 1.000 Do Not Test

42.000 vs. 24.000 4.220 11 0.811 1.000 Do Not Test

42.000 vs. 30.000 2.272 11 0.437 1.000 Do Not Test

42.000 vs. 36.000 1.299 11 0.249 1.000 Do Not Test

36.000 vs. 0.000 7.466 11 1.434 0.995 Do Not Test

36.000 vs. 6.000 6.817 11 1.310 0.998 Do Not Test

36.000 vs. 12.000 5.519 11 1.060 1.000 Do Not Test

36.000 vs. 18.000 4.220 11 0.811 1.000 Do Not Test

36.000 vs. 24.000 2.922 11 0.561 1.000 Do Not Test

36.000 vs. 30.000 0.974 11 0.187 1.000 Do Not Test

30.000 vs. 0.000 6.493 11 1.247 0.998 Do Not Test

30.000 vs. 6.000 5.843 11 1.123 0.999 Do Not Test

30.000 vs. 12.000 4.545 11 0.873 1.000 Do Not Test

30.000 vs. 18.000 3.246 11 0.624 1.000 Do Not Test

30.000 vs. 24.000 1.948 11 0.374 1.000 Do Not Test

24.000 vs. 0.000 4.545 11 0.873 1.000 Do Not Test

24.000 vs. 6.000 3.896 11 0.748 1.000 Do Not Test

24.000 vs. 12.000 2.597 11 0.499 1.000 Do Not Test

24.000 vs. 18.000 1.299 11 0.249 1.000 Do Not Test

18.000 vs. 0.000 3.246 11 0.624 1.000 Do Not Test

18.000 vs. 6.000 2.597 11 0.499 1.000 Do Not Test

18.000 vs. 12.000 1.298 11 0.249 1.000 Do Not Test

12.000 vs. 0.000 1.948 11 0.374 1.000 Do Not Test

12.000 vs. 6.000 1.299 11 0.249 1.000 Do Not Test

6.000 vs. 0.000 0.649 11 0.125 1.000 Do Not Test

Comparisons for factor: **Voltage within 0**

**Comparison Diff of Means p q P P<0.050**

7V vs. 3V 0.000 3 0.000 1.000 No

7V vs. 5V 0.000 3 0.000 1.000 Do Not Test

5V vs. 3V 0.000 3 0.000 1.000 Do Not Test

Comparisons for factor: **Voltage within 6**

**Comparison Diff of Means p q P P<0.050**

7V vs. 3V 8.765 3 1.684 0.462 No

7V vs. 5V 0.974 3 0.187 0.990 Do Not Test

5V vs. 3V 7.791 3 1.497 0.542 Do Not Test

Comparisons for factor: **Voltage within 12**

**Comparison Diff of Means p q P P<0.050**

7V vs. 3V 16.556 3 3.181 0.068 No

7V vs. 5V 0.974 3 0.187 0.990 Do Not Test

5V vs. 3V 15.582 3 2.994 0.092 Do Not Test

Comparisons for factor: **Voltage within 18**

**Comparison Diff of Means p q P P<0.050**

7V vs. 3V 24.672 3 4.740 0.003 Yes

7V vs. 5V 0.974 3 0.187 0.990 No

5V vs. 3V 23.698 3 4.553 0.005 Yes

Comparisons for factor: **Voltage within 24**

**Comparison Diff of Means p q P P<0.050**

5V vs. 3V 41.227 3 7.921 <0.001 Yes

5V vs. 7V 7.466 3 1.434 0.570 No

7V vs. 3V 33.761 3 6.486 <0.001 Yes

Comparisons for factor: **Voltage within 30**

**Comparison Diff of Means p q P P<0.050**

5V vs. 3V 49.343 3 9.480 <0.001 Yes

5V vs. 7V 7.466 3 1.434 0.570 No

7V vs. 3V 41.877 3 8.046 <0.001 Yes

Comparisons for factor: **Voltage within 36**

**Comparison Diff of Means p q P P<0.050**

7V vs. 3V 59.406 3 11.413 <0.001 Yes

7V vs. 5V 1.948 3 0.374 0.962 No

5V vs. 3V 57.459 3 11.039 <0.001 Yes

Comparisons for factor: **Voltage within 42**

**Comparison Diff of Means p q P P<0.050**

7V vs. 3V 70.444 3 13.534 <0.001 Yes

7V vs. 5V 6.493 3 1.247 0.653 No

5V vs. 3V 63.951 3 12.287 <0.001 Yes

Comparisons for factor: **Voltage within 48**

**Comparison Diff of Means p q P P<0.050**

7V vs. 3V 81.156 3 15.592 <0.001 Yes

7V vs. 5V 8.440 3 1.622 0.488 No

5V vs. 3V 72.716 3 13.971 <0.001 Yes

Comparisons for factor: **Voltage within 54**

**Comparison Diff of Means p q P P<0.050**

7V vs. 3V 94.791 3 18.212 <0.001 Yes

7V vs. 5V 20.127 3 3.867 0.020 Yes

5V vs. 3V 74.664 3 14.345 <0.001 Yes

Comparisons for factor: **Voltage within 60**

**Comparison Diff of Means p q P P<0.050**

7V vs. 3V 103.880 3 19.958 <0.001 Yes

7V vs. 5V 22.075 3 4.241 0.010 Yes

5V vs. 3V 81.806 3 15.717 <0.001 Yes

A result of "Do Not Test" occurs for a comparison when no significant difference is found between two means that enclose that comparison. For example, if you had four means sorted in order, and found no difference between means 4 vs. 2, then you would not test 4 vs. 3 and 3 vs. 2, but still test 4 vs. 1 and 3 vs. 1 (4 vs. 3 and 3 vs. 2 are enclosed by 4 vs. 2: 4 3 2 1). Note that not testing the enclosed means is a procedural rule, and a result of Do Not Test should be treated as if there is no significant difference between the means, even though one may appear to exist.

**Sustain**

**Drug Release**

**One Way Analysis of Variance** Tuesday, July 21, 2020, 3:08:31 AM

**Data source:** Data 1 in Notebook3

Dependent Variable: Fluorescence

**Normality Test (Shapiro-Wilk):**  Passed (P = 0.160)

**Equal Variance Test (Brown-Forsythe):** Passed (P = 0.124)

**Group Name N Missing Mean Std Dev SEM**

0 min 6 0 58.601 11.530 4.707

5 min 6 0 59.731 14.913 6.088

10 min 6 0 56.907 6.354 2.594

15 min 6 0 43.009 8.442 3.447

**Source of Variation DF SS MS F P**

Between Groups 3 1092.082 364.027 3.118 0.049

Residual 20 2334.841 116.742

Total 23 3426.923

The differences in the mean values among the treatment groups are greater than would be expected by chance; there is a statistically significant difference (P = 0.049).

Power of performed test with alpha = 0.050: 0.460

All Pairwise Multiple Comparison Procedures (Tukey Test):

Comparisons for factor: **Time**

**Comparison Diff of Means p q P P<0.050**

5 min vs. 15 min 16.723 4 3.791 0.064 No

5 min vs. 10 min 2.824 4 0.640 0.968 Do Not Test

5 min vs. 0 min 1.130 4 0.256 0.998 Do Not Test

0 min vs. 15 min 15.592 4 3.535 0.091 Do Not Test

0 min vs. 10 min 1.694 4 0.384 0.993 Do Not Test

10 min vs. 15 min 13.898 4 3.151 0.150 Do Not Test

A result of "Do Not Test" occurs for a comparison when no significant difference is found between two means that enclose that comparison. For example, if you had four means sorted in order, and found no difference between means 4 vs. 2, then you would not test 4 vs. 3 and 3 vs. 2, but still test 4 vs. 1 and 3 vs. 1 (4 vs. 3 and 3 vs. 2 are enclosed by 4 vs. 2: 4 3 2 1). Note that not testing the enclosed means is a procedural rule, and a result of Do Not Test should be treated as if there is no significant difference between the means, even though one may appear to exist.

**NGF**

**release**

**One Way Analysis of Variance** Tuesday, July 21, 2020, 3:27:44 AM

**Data source:** Data 1 in Notebook4

Dependent Variable: NGF

**Normality Test (Shapiro-Wilk):**  Passed (P = 0.962)

**Equal Variance Test (Brown-Forsythe):** Passed (P = 0.297)

**Group Name N Missing Mean Std Dev SEM**

rhGF 6 0 13269.333 2042.456 833.829

BiotinGF 6 0 9603.000 1968.005 803.435

ReleasedGF 6 0 8863.667 2074.068 846.735

Control 6 0 4637.667 769.891 314.307

**Source of Variation DF SS MS F P**

Between Groups 3 225626689.833 75208896.611 23.250 <0.001

Residual 20 64695814.000 3234790.700

Total 23 290322503.833

The differences in the mean values among the treatment groups are greater than would be expected by chance; there is a statistically significant difference (P = <0.001).

Power of performed test with alpha = 0.050: 1.000

All Pairwise Multiple Comparison Procedures (Tukey Test):

Comparisons for factor: **Group**

**Comparison Diff of Means p q P P<0.050**

rhGF vs. Control 8631.667 4 11.756 <0.001 Yes

rhGF vs. ReleasedGF 4405.667 4 6.000 0.002 Yes

rhGF vs. BiotinGF 3666.333 4 4.993 0.010 Yes

BiotinGF vs. Control 4965.333 4 6.762 <0.001 Yes

BiotinGF vs. ReleasedGF 739.333 4 1.007 0.891 No

ReleasedGF vs. Control 4226.000 4 5.755 0.003 Yes

**bFGF**

**release**

**One Way Analysis of Variance** Tuesday, July 21, 2020, 3:28:11 AM

**Data source:** Data 1 in Notebook4

Dependent Variable: bFGF

**Normality Test (Shapiro-Wilk):**  Passed (P = 0.999)

**Equal Variance Test (Brown-Forsythe):** Passed (P = 0.755)

**Group Name N Missing Mean Std Dev SEM**

rhGF 6 0 14840.833 743.409 303.495

BiotinGF 6 0 11894.167 1202.173 490.785

ReleasedGF 6 0 12239.500 1036.679 423.222

Control 6 0 6371.500 1012.693 413.430

**Source of Variation DF SS MS F P**

Between Groups 3 228347869.333 76115956.444 74.293 <0.001

Residual 20 20490630.667 1024531.533

Total 23 248838500.000

The differences in the mean values among the treatment groups are greater than would be expected by chance; there is a statistically significant difference (P = <0.001).

Power of performed test with alpha = 0.050: 1.000

All Pairwise Multiple Comparison Procedures (Tukey Test):

Comparisons for factor: **Group**

**Comparison Diff of Means p q P P<0.050**

rhGF vs. Control 8469.333 4 20.496 <0.001 Yes

rhGF vs. BiotinGF 2946.667 4 7.131 <0.001 Yes

rhGF vs. ReleasedGF 2601.333 4 6.295 0.001 Yes

ReleasedGF vs. Control 5868.000 4 14.200 <0.001 Yes

ReleasedGF vs. BiotinGF 345.333 4 0.836 0.934 No

BiotinGF vs. Control 5522.667 4 13.365 <0.001 Yes
