# Supplementary material for: Electrically Stimulated Tunable Drug Delivery From Polypyrrole-Coated Polyvinylidene Fluoride
Source: Front Chem. 2021 Feb 5;9:599631. doi: 10.3389/fchem.2021.599631 (PMC7892451; doi:10.3389/fchem.2021.599631)
Supplement: Supplementary file 3 [file table3.docx]

**X-ray Photoelectron Spectroscopy characterization**

1. **Methods**

X-ray Photoelectron Spectroscopy (XPS) was conducted using a K-Alpha+ surface analyzer (Thermofisher, Waltham, MA, USA) to evaluate the surface composition of the PPy coating using a AlKα source and a spot size of 400 µm at a detector pass energy of 200 eV and generating a survey spectrum from 0-1360 eV at a resolution of 1.0 eV. The detected counts per second of atoms at binding energy associated with F1s and N1s were calculated, and the relative ratio of these in the sample was tabulated as evidence of PVDF and coverage of Polypyrrole/Biotin in the PPy-coated PVDF fiber samples.

1. **Results**

X-ray Photoelectron Spectroscopy (XPS) analysis confirmed the change in chemical composition from the PVDF substrate to the PPy coated samples. The impact of polymerization time on PPy coating characteristics was best represented by the changing ratio between the percentage of F1s and N1s atoms (**Table S2**) observed from the survey spectra (**Figure S1**). N1s is only present in PPy, and changed from 0.33% (background levels) in the PVDF group to 80% in the 24 hour polymerization PPy coated group, relative to the F1s which represents the fluorine present in the PVDF (but absent in PPy) which decreases with increasing PPy coating polymerization time confirming the increase and coverage of PPy coating on the PVDF fibers.


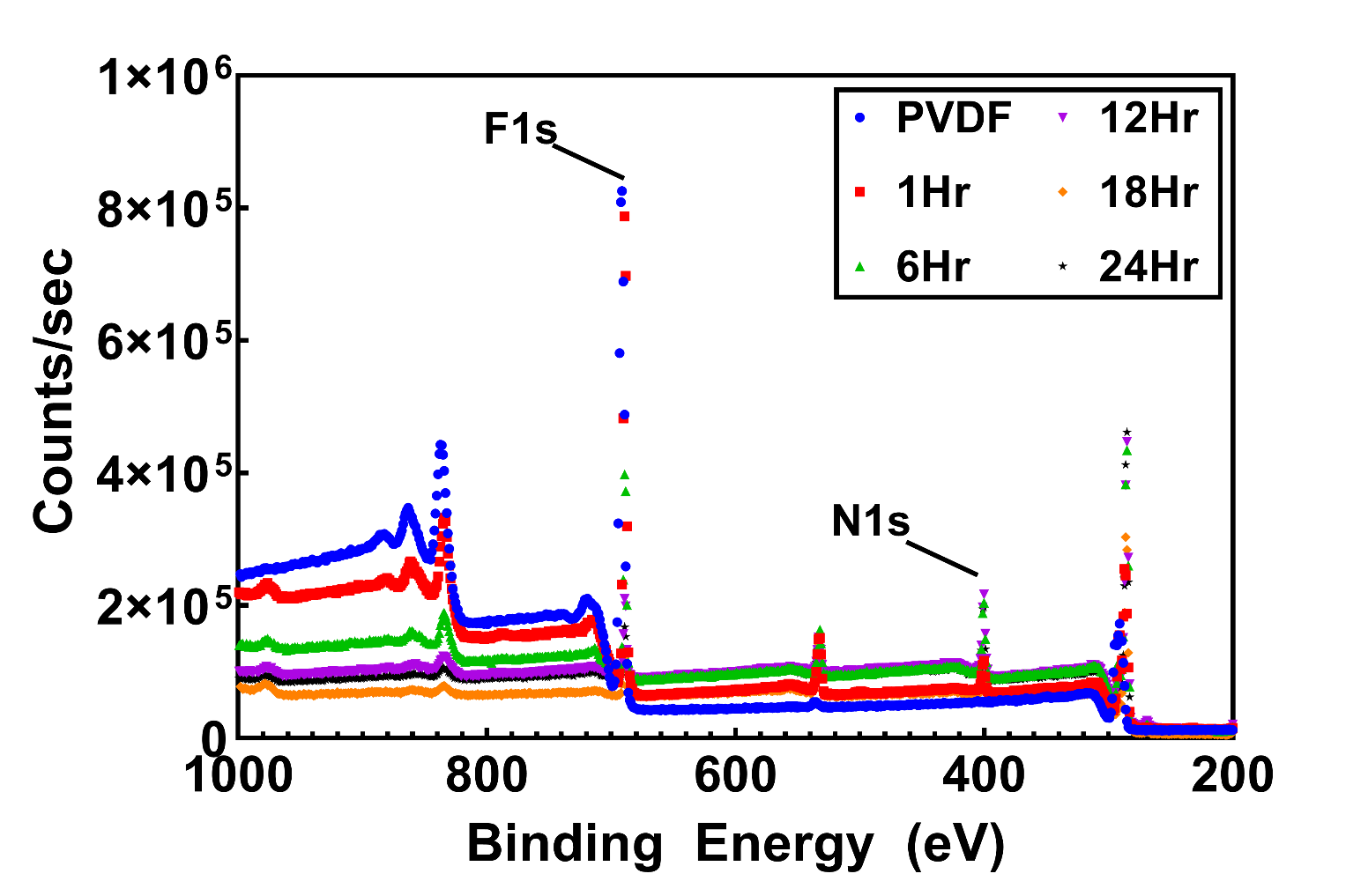


**Figure S1.** The survey spectra obtained for the base PVDF and the 5 polymerization durations of PPy coatings (1Hr, 6Hr, 12Hr, 18Hr and 24Hr) showing binding energy specific respective peaks in counts for F1s and N1s that were used to calculate the relative atomic percentages.

1. **Table S1** Results from the XPS survey spectrum of relative ratio of F1s and N1s atoms for the various samples of PVDF and PPy coated PVDF

| **Sample** | **Name** | **Atomic Percent** | **Peak BE** | **Height CPS** | **FWHM eV** | **Area (P) CPS.eV** |
| --- | --- | --- | --- | --- | --- | --- |
| **PVDF** | F1s | **99.67** | 691.25 | 761244 | 4.74 | 3804223 |
|  | N1s | **0.33** | 405.08 | 1829.82 | 0.1 | 6443.27 |
| **1Hr** | F1s | **83.24** | 688.72 | 520235.7 | 1.58 | 1507281 |
|  | N1s | **16.76** | 400.42 | 42002.95 | 3.26 | 155829.8 |
| **6Hr** | F1s | **50.43** | 688.68 | 235322.7 | 1.43 | 679520.5 |
|  | N1s | **49.57** | 400.25 | 98144.47 | 2.95 | 343047.6 |
| **12Hr** | F1s | **25.54** | 688.7 | 88195.22 | 1.55 | 258091.8 |
|  | N1s | **74.46** | 400.23 | 107081.2 | 2.97 | 386400.5 |
| **18Hr** | F1s | **22.72** | 688.88 | 38205.42 | 2.51 | 106237.2 |
|  | N1s | **77.28** | 400.83 | 55971.58 | 2.93 | 185513.2 |
| **24Hr** | F1s | **19.9** | 688.77 | 57163.59 | 1.6 | 157570.5 |
|  | N1s | **80.1** | 400.35 | 98210.11 | 2.75 | 325626.8 |

**Table S2.** Elemental composition of PVDF and PPy coated (for various durations from 1 hour to 24 hours) PVDF aligned electrospun fibers, determined using XPS analysis.

| Sample | **PVDF** | **1hr** | **6hrs** | **12hrs** | **18hrs** | **24hrs** |
| --- | --- | --- | --- | --- | --- | --- |
| **F1s %** | 99.67 | 83.24 | 50.43 | 25.54 | 22.72 | 19.9 |
| **N1s %** | 0.33 | 16.76 | 49.57 | 74.46 | 77.28 | 80.1 |
