# Supplementary figures and images for: Electrically Stimulated Tunable Drug Delivery From Polypyrrole-Coated Polyvinylidene Fluoride
Source: Front Chem. 2021 Feb 5;9:599631. doi: 10.3389/fchem.2021.599631 (PMC7892451; doi:10.3389/fchem.2021.599631)

1Hr PPy

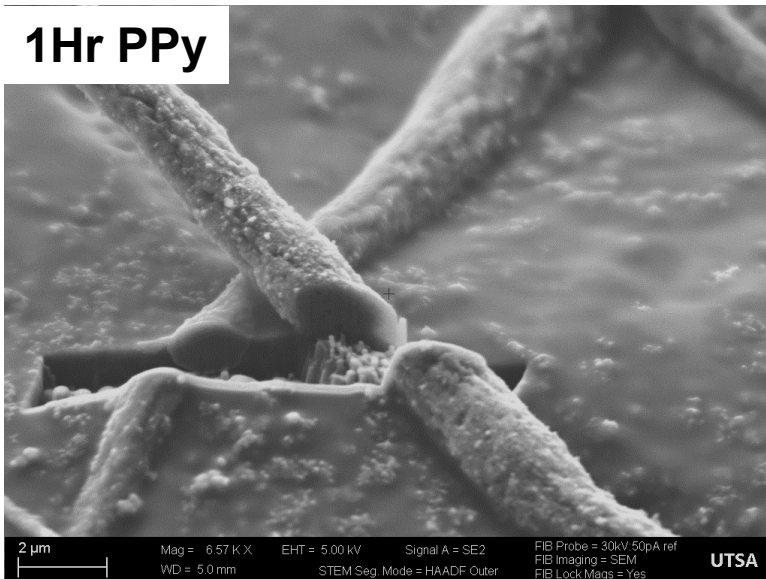

6Hr PPy

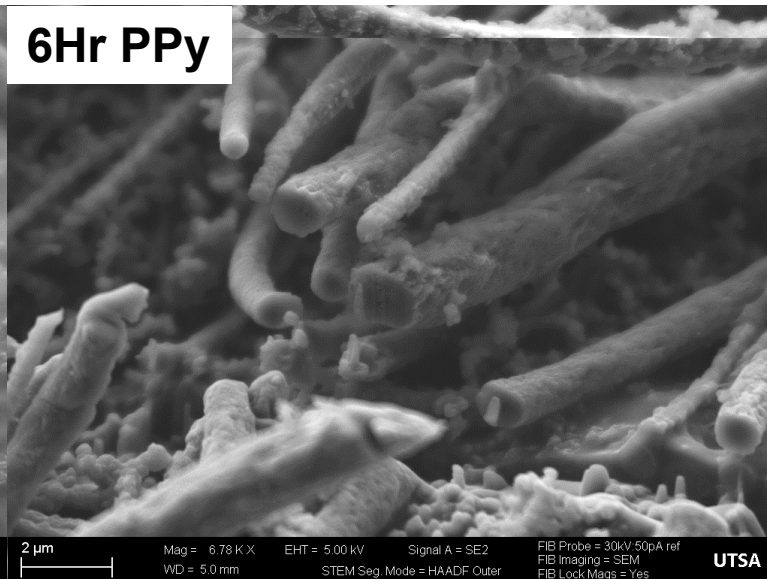

12Hr PPy

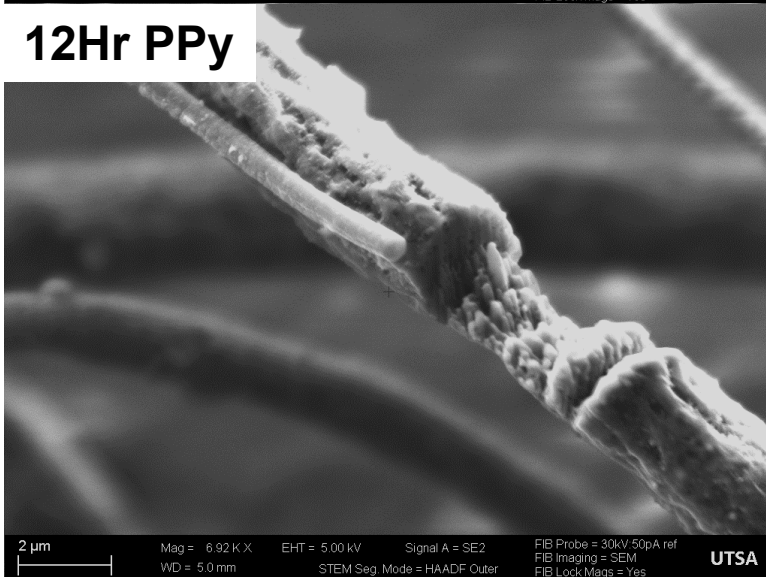

18Hr PPy

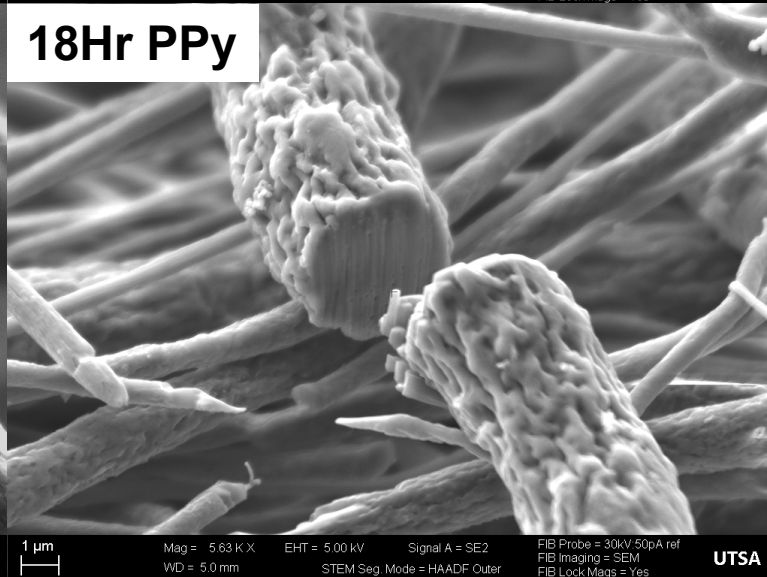

24Hr PPy

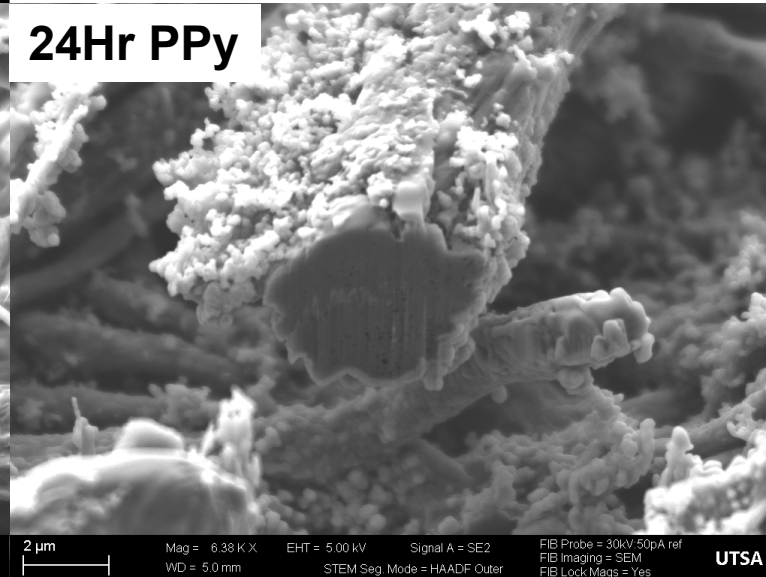

Supplement: Supplementary file 4 [file datasheet1.pdf]

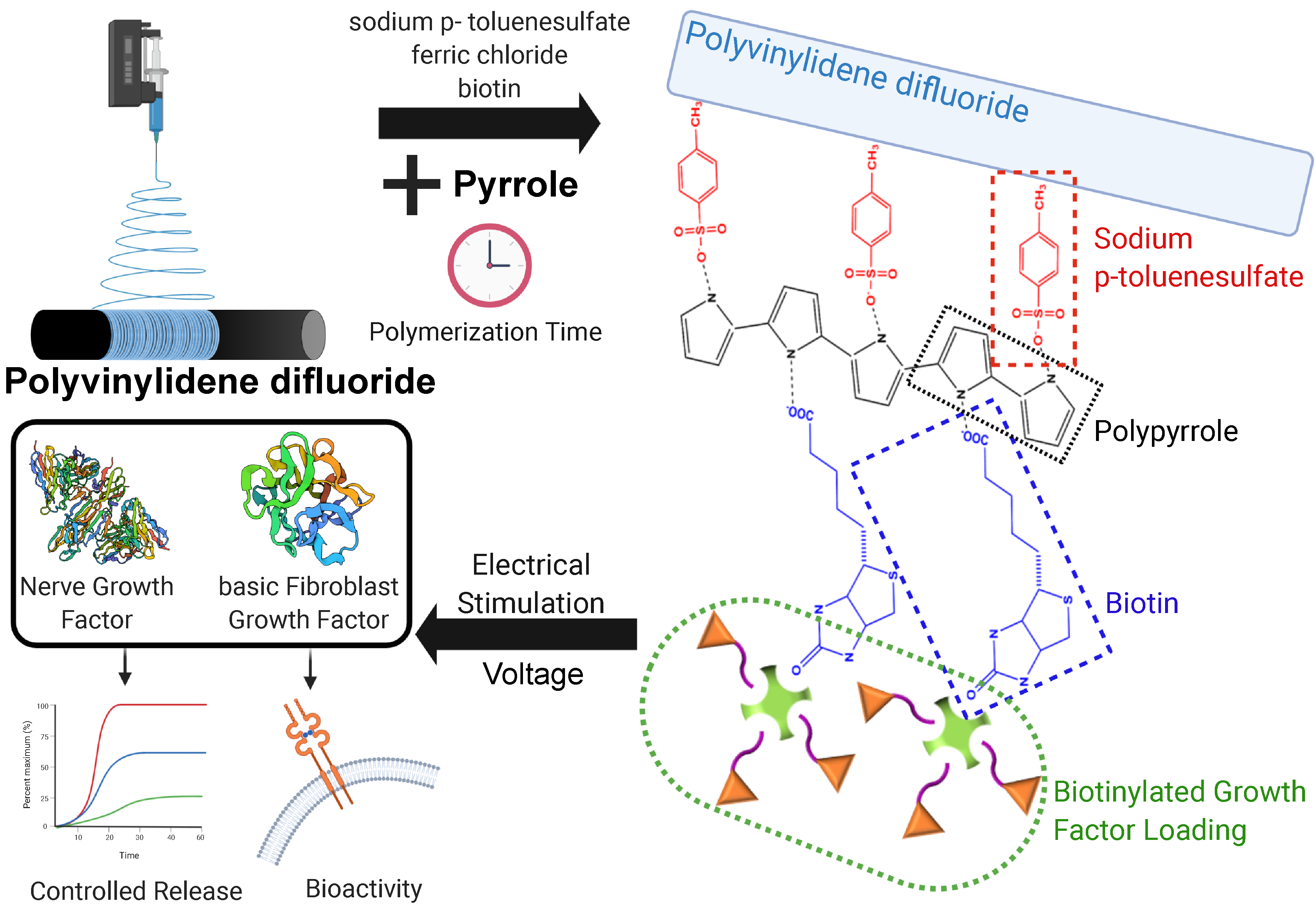

Supplement: Supplementary file 5 [file image1.tif]
